# A Taxon-Wise Insight Into Rock Weathering and Nitrogen Fixation Functional Profiles of Proglacial Systems

**DOI:** 10.3389/fmicb.2021.627437

**Published:** 2021-09-16

**Authors:** Gilda Varliero, Alexandre M. Anesio, Gary L. A. Barker

**Affiliations:** ^1^School of Biological Sciences, University of Bristol, Bristol, United Kingdom; ^2^Department of Environmental Sciences, Aarhus University, Roskilde, Denmark

**Keywords:** forefield, soil, microbial succession, rock weathering, nitrogen fixation, functional profiles, microbial diversity

## Abstract

The Arctic environment is particularly affected by global warming, and a clear trend of the ice retreat is observed worldwide. In proglacial systems, the newly exposed terrain represents different environmental and nutrient conditions compared to later soil stages. Therefore, proglacial systems show several environmental gradients along the soil succession where microorganisms are active protagonists of the soil and carbon pool formation through nitrogen fixation and rock weathering. We studied the microbial succession of three Arctic proglacial systems located in Svalbard (Midtre Lovénbreen), Sweden (Storglaciären), and Greenland (foreland close to Kangerlussuaq). We analyzed 65 whole shotgun metagenomic soil samples for a total of more than 400 Gb of sequencing data. Microbial succession showed common trends typical of proglacial systems with increasing diversity observed along the forefield chronosequence. Microbial trends were explained by the distance from the ice edge in the Midtre Lovénbreen and Storglaciären forefields and by total nitrogen (TN) and total organic carbon (TOC) in the Greenland proglacial system. Furthermore, we focused specifically on genes associated with nitrogen fixation and biotic rock weathering processes, such as nitrogenase genes, *obcA* genes, and genes involved in cyanide and siderophore synthesis and transport. Whereas we confirmed the presence of these genes in known nitrogen-fixing and/or rock weathering organisms (e.g., *Nostoc*, *Burkholderia*), in this study, we also detected organisms that, even if often found in soil and proglacial systems, have never been related to nitrogen-fixing or rock weathering processes before (e.g., *Fimbriiglobus*, *Streptomyces*). The different genera showed different gene trends within and among the studied systems, indicating a community constituted by a plurality of organisms involved in nitrogen fixation and biotic rock weathering, and where the latter were driven by different organisms at different soil succession stages.

## Introduction

Due to global warming, a clear trend of glacial ice retreat has been observed in recent decades worldwide (Moon et al., 2018; Maurer et al., 2019). This rapid loss of the cryosphere is leading to an expansion of proglacial systems, exposing bedrocks that have been covered by ice for thousands of years (Fountain et al., 2012; Heckmann et al., 2016). Chemical, physical, and biological processes lead to the formation of soil from the mineralization of the bedrocks during weathering (Uroz et al., 2009, 2015; Xi et al., 2018). Biological rock weathering is a key process in environments where soil microbiota and vegetation roots help the release of nutrients and major ions into the soil with their metabolism and mechanical actions (Kelly et al., 1998; Borin et al., 2010; Porder, 2019). The release of nutrients and major ions represents a source of enzyme cofactors and energy for the soil microbiota, especially in nutrient-poor soils, such as the early stage glacial forefield soils (Uroz et al., 2015), giving microbes a pivotal role in soil formation (Rousk and Bengtson, 2014; Koshila Ravi et al., 2019). Furthermore, other key environmental and ecological processes, such as nitrogen fixation, take place in proglacial systems. These environments are habitats for diverse diazotrophic communities that, using the enzyme nitrogenase, progressively enrich the soil with ammonia and bioavailable nitrogen sources to non-diazotrophic organisms (Bradley et al., 2014; Nash et al., 2018).

Rock weathering and nitrogen fixation create gradient conditions in the proglacial environments. Whereas the ground in the ice margins is dominated by rocks, the soil content increases and deepens going farther away from it, with an associated increase in vegetation. The bioavailability of nutrients, such as organic carbon and nitrogen, also increases with the formation of the soil. The presence of such gradients and the progressive ground exposure to the atmosphere make proglacial systems very suitable for the study of microbial succession (Edwards and Cook, 2015). Microbial communities show trends along chronosequences (Schmalenberger and Noll, 2010; Zumsteg et al., 2012; Bajerski and Wagner, 2013; Jiang et al., 2019b). Previous work has shown that the microbial communities close to the ice edge are usually dominated by autotrophs and chemolithotrophs that are able to use soil minerals and sunlight as energy source and enrich the soil with biological available organic carbon and nutrients (Schmidt et al., 2008; Liu et al., 2012; Fernández-Martínez et al., 2017). These first stages of the succession are also the most influenced by the glacier inputs and discharges in the environment (Hotaling et al., 2017). Going farther from the ice, different studies have observed a decrease of the autotroph component and an increase in the heterotroph microbial component, where the latter takes advantage of the progressive organic-enriched soil (Bradley et al., 2016). These trends are also accompanied by an increase of the vegetation complexity with the distance from the ice edge, establishing also symbiotic and mutualistic relationships with the soil microbiome (Knelman et al., 2012; Rime et al., 2016).

Despite the pivotal role of microbial communities in rock weathering, the protagonists and the mechanisms of these processes are not very clear. Different mechanisms and rock weathering-enhancing organisms have been reported in studies of soil isolates of both bacteria (Frey et al., 2010; Lepleux et al., 2012; Liu et al., 2012; Olsson-Francis et al., 2015; Xi et al., 2018; Wang et al., 2019) and fungi (Brunner et al., 2011), observing the production of organic acids (e.g., oxalate) and hydrogen cyanide (HCN) to mobilize the nutrients such as iron sulfur and phosphorus (El-Tarabily et al., 2008), and an increase in siderophore production to import iron into the cell (Frey et al., 2010; Wongfun et al., 2014; Olsson-Francis et al., 2015). Compared to rock weathering processes, diazotrophic organisms are better understood and characterized. Organisms spanning more than 13 phyla have been identified as nitrogen fixers (Addo and Dos Santos, 2020). Diazotrophic assemblages vary in relation to the soil and rhizosphere characteristics (Duc et al., 2009) and are more abundant in the first stages of the forefield succession where they enrich the soil with nitrogen, a key nutrient for cellular growth (Brankatschk et al., 2011). Despite the importance of this process in forefield dynamics, there is a lack of understanding of the diazotrophic organism variation along the proglacial microbial succession (Brankatschk et al., 2011; Nash et al., 2018).

Whereas previous studies have analyzed forefield microbial communities with several approaches, such as 16S rRNA gene sequencing (e.g., Bajerski and Wagner, 2013; Fernández-Martínez et al., 2017), clone library sequencing (e.g., Zumsteg et al., 2012), GeoChip microarray (Fernández-Martínez et al., 2016), and ecoenzymatic stoichiometry (Jiang et al., 2019a; Li et al., 2020). Only a few studies use a whole shotgun metagenomic sequencing approach (e.g., Nash et al., 2018); none of these, however, report taxon-wise functional profiles along forefield successions. We reanalyzed the whole shotgun sequencing dataset reported in Nash et al. (2018) to obtain a comprehensive picture of microbial diversity and gene function over glacial chronosequences. In particular, we analyzed 65 different metagenomes from three different proglacial systems: two forefields from two small glacier valleys, the Midtre Lovénbreen in Svalbard and the Storglaciären in Sweden, and a proglacial field of the Greenland ice sheet (GrIS) in proximity of point 601. In this work, we report (i) how taxonomical groups varied along the different proglacial successional gradients and (ii) which organisms were involved in different proglacial processes. We focused on two of the processes that shape forefield dynamics and nutrient bioavailability the most: nitrogen fixation, exploring nitrogenase genes, and rock weathering processes, looking at the *obcA* genes that are involved in the first step of oxalate biosynthesis (Nakata, 2011), genes involved in cyanide synthesis, and genes involved in siderophore synthesis and transport.

## Materials and Methods

This dataset was previously analyzed in Nash et al. (2018) to show diazotrophic community variations among the three proglacial systems and in relation to the measured total nitrogen (TN) and total organic carbon (TOC) concentrations for each of the systems. However, no microbial succession along the different proglacial systems was analyzed in Nash et al. (2018). Therefore, we reanalyzed the dataset (i) to obtain information on relevant microbial successional patterns observed in the studied glacial forefields and (ii) to study the functional patterns related to proglacial soil formation focusing on not only nitrogen fixation but also rock weathering phenomena (Table 1).

**TABLE 1:** Comparative table on the main aspects of Nash et al. (2018) and this study.

	Study aspects	Nash et al., 2018	This study
**Metagenomic soil samples**	Number of samples	70	65
	Studied proglacial systems	Midtre Lovénbreen, Storglaciären, and Rabots glacier forefields; and the proglacial field of the Greenland ice sheet in proximity of point 601	Midtre Lovénbreen and Storglaciären forefields; and the proglacial field of the Greenland ice sheet in proximity of point 601 (i.e., same samples as collected, and sequenced from Nash et al., 2018)
**Geochemistry**	Analyzed variables	TOC and TN	TOC and TN (data from Nash et al., 2018)
**Bioinformatics**	Read assembly	SPADES on each forefield dataset separately	MEGAHIT on all forefield datasets together
	Gene and taxon annotation	BLAST + MEGAN	Diamond + LongMeta
**Study aims**	Taxon and functional association with soil developmental stages	No	Yes
	Functional characterization	*nif* genes	Functional trends along different developmental soil stages with a focus on genes involved in nitrogen fixation (i.e., *nif* genes) and rock weathering phenomena (i.e., *obcA* genes, genes involved in cyanide and siderophore synthesis)
	Taxon characterization	Diazotrophs present in the different proglacial systems	Taxonomic trends along different developmental soil stages

*TN, total nitrogen; TOC, total organic carbon.*

Sample collection, soil geochemical characterization, sample preparation, and sequencing methods are reported in Nash et al. (2018). We briefly report details about sampling and forefield characterization in *Site Characterization*.

### Site Characterization

**FIGURE 1:**
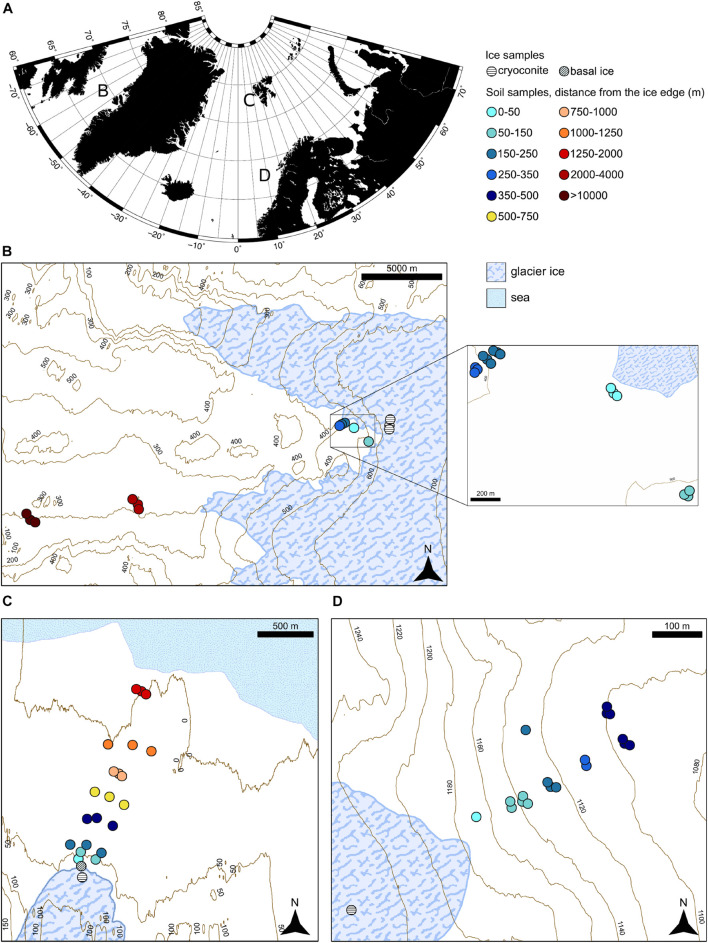
Sampling site locations. **(A)** Overview of the sampling site. **(B)** Greenland forefield sampling site. **(C)** Svalbard forefield sampling site. **(D)** Sweden forefield sampling site.

Samples were collected from the Midtre Lovénbreen forefield during summer 2013 and from the Storglaciären forefield and the proglacial field of the GrIS in proximity of point 601 during summer 2014. The sampling was conducted with the same approach in all the systems: only the top 10 cm of the soil was collected in a sterile Whirl-pack bag (Whirl-Pak, Nasco, United States) and then frozen at −20°C until processing. For Midtre Lovénbreen and Storglaciären, samples were transferred to −20°C storage on the day of collection, whereas the remote location of the Greenland field sites meant that samples were stored on ice in a cool box for up to 2 days before returning to the freezer at the Kangerlussuaq International Science Station. The soil samples were collected along transects starting in the proximity of the ice edge, going farther away. Where possible, multiple samples were taken at the same distance from the ice edge. Samples were not collected close to vegetation patches, rivers, or discontinuous soil patches to minimize site-specific effects. Samples were collected from three different proglacial systems sited in the Arctic circle (Nash et al., 2018; Figure 1A). These systems present different morphologies and have a different deglaciation rate, the ice sheet being much slower than the glaciers in the small valleys and the collected soil having different ages since deglaciation. The sampling size area and the geographical characteristics in the three different systems were also considerably different. The samples in Greenland were taken up to 10 km away from the closest ice point (Figure 1B). The samples in Svalbard were taken up to 1,600 m from the glacier toe and, as the forefield faces a fjord, this last point of the succession is sited close to the seawater (Figure 1C). Samples in the forefield from Sweden were taken up to a river that delimits the end of the small forefield area, at 350 m from the ice margin (Figure 1D). This diversity and the geographical dispersion of these sites allowed to compare functional and taxonomical trends between different systems. Even if the three forefield successions span different soil distances from the ice edge and different soil developmental stages, the generic comparison between young and older soils in different chronosequences (even if they represent different age increments) still provides insights into overall trends in soil development. The Greenland proglacial system is referred to here as G, while Svalbard is referred to as SV, and the forefield in Sweden as SW.

The samples were classified by distance from the glacier toe, and the distance was calculated as the distance between the sampling site and the closest ice edge point. Each sample was also characterized by TN and TOC values. The latter were measured by Nash et al. (2018) using mass spectrometry and carbon elemental analyzer.

### Bioinformatics Analysis

A total of 65 samples were collected and Illumina whole shogun sequenced (Nash et al., 2018). The Illumina reads were checked, and quality was trimmed with FastQC v 0.11.7^1^ and Trimmomatic v 0.36 (Bolger et al., 2014). The latter was run with the command options ILLUMINACLIP:TruSeq2-PE.fa:2:30:10 MINLEN:26, and the additional options TRAILING:10 for SV, SLIDINGWINDOW:5:20 CROP:149 for SW, and SLIDINGWINDOW:5:24 MINLEN:26 CROP:149 for G. The quality-checked sequences were then co-assembled from the three different datasets with the software MEGAHIT v 1.1.3 (Li et al., 2015) and parameters –k-min 25 –k-max 145 –k-step 8 -t 64.

Only assembly contigs longer than 300 bp were retrieved and aligned against the NCBI non-redundant (nr) protein database v 5 (Sayers et al., 2020) with Diamond 0.9.22 (Buchfink et al., 2015) using the command line options -e 0.000001 -F 15 –range-culling –range-cover 20 –id 50 –top 10 -f 6 -c1 -b4.0.

LongMeta, a custom pipeline whose scripts are publicly available on GitHub^2^, was used to detect and split chimeric contigs and to assign taxonomy and gene-coding regions to the assembly using the known protein alignment information. Functionality information was then associated with both nr (Sayers et al., 2020) and Gene Ontology (GO) nomenclature (Ashburner et al., 2000; Carbon et al., 2019). Reads were mapped back to the assembly with Bowtie2 v 2.3.4.3 (Langmead and Salzberg, 2012) and parameters –phred33 –local -I 100 -X 800 –no-hd –no-unal -D 30 -R 3 -N 0 -L 20 -i S,1,0.25 –non-deterministic -p 20. LongMeta was then used to assign taxonomic and function abundance information to each sample. LongMeta outputs taxonomy and functionality data as base coverage. In this article, whereas gene information is reported as base coverage, taxonomy information is reported as relative abundance (calculated from base coverage).

### Statistical Analysis

The geochemical dataset and LongMeta abundance and coverage datasets were imported to the R environment (R Core Team 2019, 2019) where all the statistical analyses were performed. In all the graphical visualizations and the principal component analysis (PCA) (Abdi and Williams, 2010), the samples were categorized in relation to their distances from the ice edge and divided into different groups: 0–50, 50–150, 150–250, 250–350, 350–500, 500–750, 750–1,000, 1,000–1,250, 1,250–2,000, 2,000–4,000, and >10,000 m (Supplementary Table 1).

Diversity indices were calculated on genus-level base coverage values with Shannon’s (H) and inverse Simpson’s (1/D) diversity indices. The diversity values were fitted with locally weighted scatterplot smoothing (LOWESS) curves in order to detect diversity trends in relation to the site distance from the ice edge.

Functional data were reported and analyzed as weighted gene coverage. The coverage of a specific gene in a specific sample was divided by the base coverage of the sample. The coverage scaling was necessary because different samples assembled with different efficiencies depending on their sequencing depth and community complexity. This led to a differential base coverage in different samples, and therefore, the gene coverages, if not scaled, could reflect the sample coverage and not the real gene coverage in the microbial community. Here, in addition to the gene coverage, functional profiles as GO biological categories are also presented.

Permutational multivariate analysis of variance (PERMANOVA) (Anderson, 2017) was performed with 9,999 permutations on Bray–Curtis dissimilarity matrices for the taxonomy, diversity, and functional datasets and Euclidean distance matrices for the geochemical dataset. The statistical tests were performed against the fixed factor Forefield that has three levels: Greenland, Svalbard, and Sweden. In order to show how the four different datasets varied with the sample distance from the ice edge and in relation to the geochemical dataset (i.e., TN and TOC), Mantel test statistic *r* using 9,999 permutations and the Spearman’s rank correlation coefficient were calculated (Smouse et al., 1986). Mantel tests were conducted on either Bray–Curtis or Euclidean matrices as for the PERMANOVA. Whereas the Mantel test was used to compare two different datasets (i.e., two different sets of variables), the Spearman’s rank correlation coefficient *r*_*s*_ (with 9,999 permutations) was directly calculated when compared with only two different variables (e.g., a specific phylum vs. distance from the ice edge). A linear model calculation was used only when comparing two variables with the same units (i.e., Proteobacteria vs. Actinobacteria relative coverage). All the statistical analyses reported in the main text were performed without the inclusion of ice samples, unless otherwise specified. Statistical tests were interpreted as significant if *p*-value < 0.05.

Several R packages were used to perform statistical analyses and plot the graphs: vegan v 2.5.6 (Oksanen, 2017), ggpubr v 0.3.0 (Kassambara, 2018), ggplot2 v 3.3.0 (Wickham, 2016), gplots v 3.0.3 (Warnes, 2012), reshape v 0.8.8 (Wickham, 2007), and gridExtra v 2.3 (Auguie, 2017).

## Results

### Geochemical Data

**FIGURE 2:**
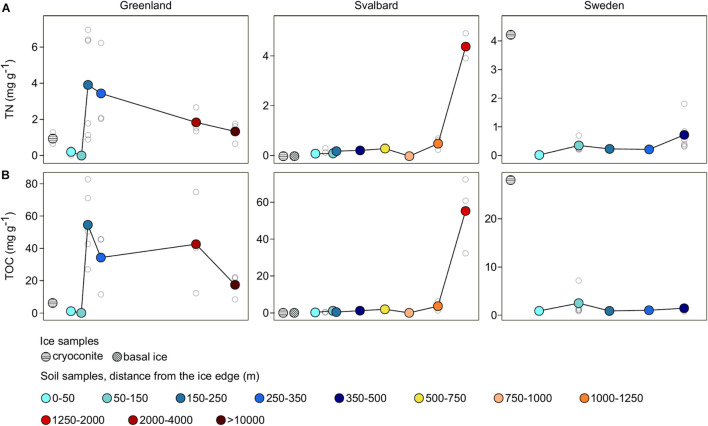
Total nitrogen (TN) **(A)** and total organic carbon (TOC) **(B)** trends across the three different proglacial systems. Samples were grouped into different categories and colored in relation to sample distance from the ice edge. Colored dots indicate the average values for each distance, whereas white dots are the values of the individual samples.

Total nitrogen and TOC showed different trends among the three proglacial systems (Figure 2). The Greenland (G) dataset showed low values in the first stages of the succession, maximum values at 150–250 m, and a gradual decrease going farther away from the glacier. The Svalbard (SV) and Swedish (SW) datasets showed an increase from the ice edge to more distant samples.

The SW dataset showed the lowest TN and TOC concentrations with values 0–2 and 0–7 mg g^–1^ in all the soil samples. The SV had a similar value range with 0–1 and 0–6 mg g^–1^, respectively, in all the soil samples except from the SV samples collected at 1,650 m from the ice edge (the closest samples to the sea; Figure 1C) where the TN and TOC mean values were 4 and 55 mg g^–1^. Compared to the SV and SW systems, G showed the highest TN and TOC values, ranging 0–7 and 0–82 mg g^–1^.

The two variables, TN and TOC, showed a positive Spearman’s correlation across all the systems (*r*_*s*_ = 0.89; *p*-value < 0.05). Sample separation among the different forefield systems (factor Forefield) explained 26% of the observed variance in this dataset (*p*-value < 0.05; Table 2A). The correlation between these two variables and the distance from the ice edge was then explored with a Mantel test statistic *r* that was equal to 0.22 (*p*-value < 0.05). Different *r* values were obtained when Mantel tests were performed on the different forefield datasets: correlation with the sample distance from the ice edge was significant only in the SV and SW forefields with an *r* of 0.39 and 0.33, respectively (*p*-value < 0.05; Table 2B).

**TABLE 2:** PERMANOVA performed between the distance from the ice edge and the geochemical dataset **(A)**. Mantel test performed to calculate the correlation between the distance from the ice edge and the geochemical dataset (i.e., TN + TOC) for the samples from the three different proglacial systems (G + SV + SW), only the G system, only the SV system, and only the SW system **(B)**. The symbol ^∗^ is reported for significant *R*^2^ and *r* values where the statistic *p*-value < 0.05.

A	*R* ^2^		*p*-value
	0.26		0.00*

**B**	**Forefield**	***r***	***p*-value**

	G + SV + SW	0.22	0.01*
	G	0.04	0.27
	SV	0.39	0.00*
	SW	0.33	0.00*

*G, Greenland; PERMANOVA, permutational multivariate analysis of variance; SV, Svalbard; SW, Sweden; TN, total nitrogen; TOC, total organic carbon.*

### Assembly Specifics

The quality-checked reads that were used to perform assembly were between 4 and 119 million (Supplementary Table 1) with 433 billion bases (433 Gb) used in total. The assembly was 30 Gb with an N50 length of 841 bases, minimum contig length of 300 bases, and maximum contig length of 561,967 bases. Reads mapped back to the assembly with a higher efficiency for ice samples (93–94%, 75–93%, and 92% for the G, SV, and SW datasets, respectively) compared to soil samples (50–78%, 34–64%, and 65–79%) (Supplementary Table 1). The average base coverage of the assembly ranged between 0.3× and 1.5× in the samples from the SV dataset, 0.7× and 3.7× in the SW dataset, and 0.7× and 3.4× in the G dataset. In each dataset, the highest coverages were observed in the ice samples. In the forefield assembly, 1% of the contigs were defined as chimeric and therefore split into shorter contigs.

### Taxonomy Diversity and Trends

Here, 1/D ranged between 25 and 128 across all the samples, whereas H ranged between 4 and 5. In all the proglacial systems, both indices were lower in the ice samples where 1/D reached a maximum of 54 and H a maximum of 4.4. Both the diversity indices had overall lower values in SV compared to SW and G datasets. However, PERMANOVA performed on the diversity dataset (i.e., 1/D and H) showed that only 20% of the variance was explained by the factor Forefield (*p*-value < 0.05) (Table 3A).

**TABLE 3:** PERMANOVA performed between the distance from the ice edge and the diversity index dataset **(A)**; taxonomy dataset at the phylum, order, and genus level **(B)**; gene dataset **(C)**; and the GO dataset **(D)**. Mantel test performed to calculate the correlation between the distance from the ice edge (distance) and the geochemical dataset (i.e., TN + TOC) with the diversity index dataset **(E)**, taxonomy dataset at the genus level **(F)**, gene dataset **(G)**, and the GO dataset **(H)**. Each of these four datasets were tested with all the samples from the three different proglacial systems (G + SV + SW), only the G system, only the SV system, and only the SW system. The symbol * is reported for significant *R*^2^ and *r* values where the statistic *p*-value < 0.05.

A		Diversity indices			
		*R* ^2^			*p*-value
		0.20			0.00*

**B**	**Taxonomy**				
	**Rank**		***R* ^2^**		***p*-value**

	Phylum		0.13		0.00*
	Order		0.22		0.00*
	Genus		0.23		0.00*

**C**	**Gene**				
		***R* ^2^**			***p*-value**

		0.26			0.00*

**D**		**GO categories**			
		***R* ^2^**			***p*-value**

		0.10			0.00*

**E**	**Diversity indices**				
	**Forefield**	**Distance**		**TN + TOC**	
		***r***	***p*-value**	***r***	***p*-value**

	G + SV + SW	–0.08	0.95	–0.13	0.98
	G	0.07	0.17	0.10	0.08
	SV	0.36	0.00*	–0.03	0.53
	SW	0.02	0.24	–0.10	0.70

**F**	**Taxonomy**				
	**Forefield**	**Distance**		**TN + TOC**	
		***r***	***p*-value**	***r***	***p*-value**

	G + SV + SW	0.04	0.25	–0.02	0.59
	G	0.19	0.04*	0.42	0.00*
	SV	0.61	0.00*	0.13	0.18
	SW	0.24	0.01*	0.01	0.41

**G**	**Gene**				
	**Forefield**	**Distance**		**TN + TOC**	
		***r***	***p*-value**	***r***	***p*-value**

	G + SV + SW	0.20	0.00*	–0.07	0.83
	G	0.20	0.07	0.29	0.01*
	SV	0.47	0.00*	0.18	0.14
	SW	0.37	0.00*	0.08	0.28

**H**	**GO categories**				
	**Forefield**	**Distance**		**TN + TOC**	
		***r***	***p*-value**	***r***	***p*-value**

	G + SV + SW	0.02	0.37	–0.18	0.98
	G	0.50	0.00*	0.07	0.20
	SV	0.31	0.00*	–0.01	0.50
	SW	0.15	0.06	–0.09	0.65

*G, Greenland; GO, gene ontology; PERMANOVA, permutational multivariate analysis of variance; SV, Svalbard; SW, Sweden; TN, total nitrogen; TOC, total organic carbon.*

**FIGURE 3:**
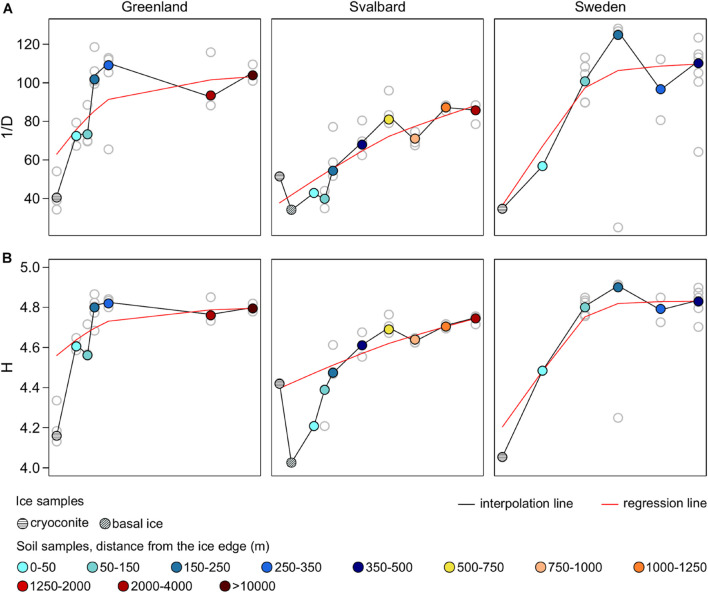
Inverse Simpson’s **(A)** and Shannon’s **(B)** diversity indices (1/D and H) calculated on the genus-level taxonomic dataset.

Fitted LOWESS lines showed increasing diversity trends along all the forefields (Figure 3). Diversity indices did not show a correlation with the distance from the ice edge (nor with TOC or TN concentration values). However, when performed on the different forefield datasets, the Mantel test statistic *r* was significant (*p*-value < 0.05) for the SV dataset (*r* = 0.36) (Table 3E). When ice samples were included together with the forefield samples, it was significant for all the datasets, where the *r* for G was 0.20, for SV was 0.45, and for SW was 0.18 (Supplementary Table 2E).

**FIGURE 4:**
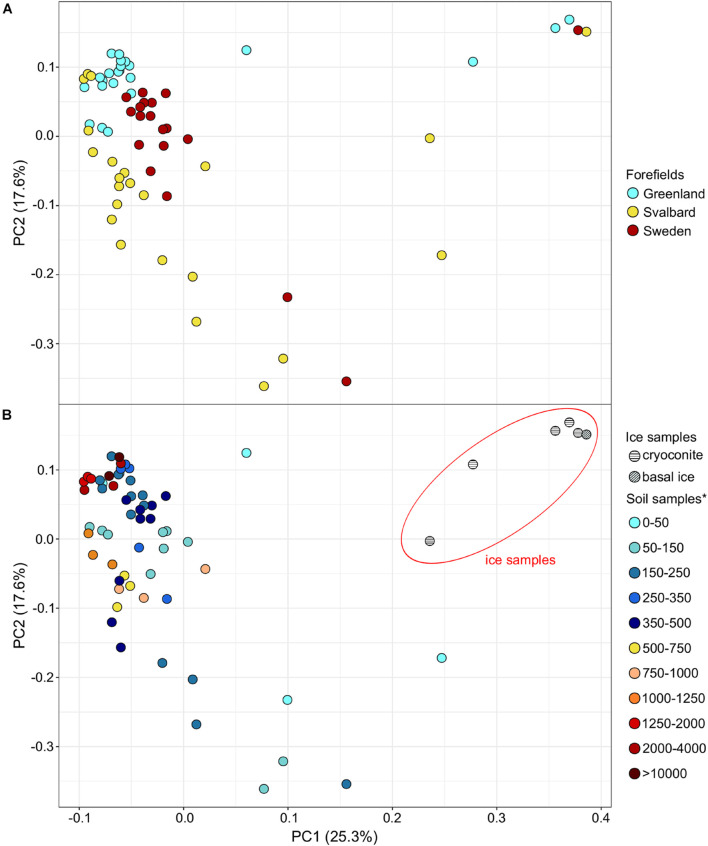
Principal component analysis (PCA) showing the sample distribution based on the genus taxonomic dataset for the forefield color-based samples **(A)** and ice and soil samples **(B)**. *Soil samples are categorized in relation to their distance from the ice edge (m).

Microbial communities showed a minor sample clustering distribution across different forefields (Figure 4A). Ice and ice edge samples (<50 m from the glacier) clearly separated from the soil samples in the PCA representation, whereas soil samples collected at different distances from the ice edge did not show clear distinct clusters between each other (Figure 4B).

Permutational multivariate analysis of variance performed on the taxonomy dataset was significant (*p*-value < 0.05) for the factor Forefield that explained the 13, 22, and 23% of the variance at phylum, order, and genus levels (Table 3B). Less variance was explained when the PERMANOVA was performed including the ice samples (Supplementary Table 2B). The Mantel test performed between distance and taxonomical dataset (genus level) was not significant. When the Mantel test statistic *r* was calculated on separate forefield datasets (i.e., G, SV, and SW), the variable distance showed a significant (*p*-value < 0.05) *r* of 0.19, 0.61, and 0.24 for G, SV, and SW, respectively (Table 3F). Only the G dataset showed a significant correlation to the geochemical dataset (i.e., TN and TOC) with *r* equal to 0.42 (Table 3F). Supplementary Figure 1 reports how the taxonomic dataset (genus level) is influenced by the variables Distance, TN, and TOC. Ice communities and early stage soil communities were mainly influenced by the genera *Thiobacillus*, *Purpureocillium*, *Methylotenera*, and *Cryobacterium*, whereas samples sited more distant in the microbial succession were conditioned by *Solirubribacter*, *Hyphomicrobium*, *Chthoniobacter*, and *Mycolicibacterium*. TOC and TN shaped the distribution of several genera, such as *Pseudolabrys*, *Bradyrhizobium*, and *Rhodoplanes*.

**FIGURE 5:**
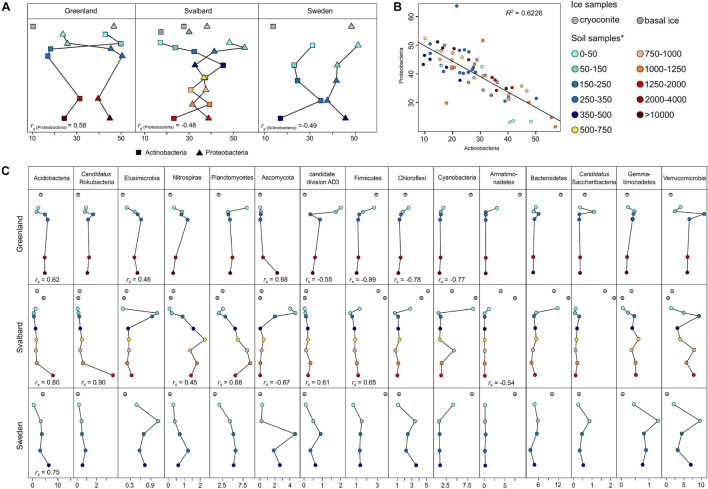
Taxonomy classification at the phylum level. **(A)** Actinobacteria and Proteobacteria trends in the dataset. **(B)** Linear model relation between Actinobacteria and Proteobacteria relative abundances (*p*-value = 3 × 10^– 15^). **(C)** Remaining phylum trends in the datasets. Spearman’s rank correlation coefficient (*r*_*s*_) was calculated between the sample distance and the relative abundance data for each phylum. *r*_*s*_ is reported below the appropriate line plot only when significant (*p*-value < 0.05). *Soil samples are categorized in relation to their distance from the ice edge (m).

At the phylum level, the two most abundant phyla, representing between 58 and 84% in the soil and 47 and 62% in the ice samples, were Proteobacteria and Actinobacteria, respectively. In all the proglacial system communities, these two phyla showed opposite trends in their abundance, especially in the latest stages of the succession (Figure 5A). The linear model showed a significant negative correlation (*R*^2^ = 0.62) between Proteobacteria and Actinobacteria (Figure 5B). The highest correlation was between Alphaproteobacteria and Actinobacteria at class level (Supplementary Table 3).

The phyla Acidobacteria, *Candidatus* Rokubacteria, Elusimicrobia, Nitrospirae, and Planctomycetes showed a significant positive Spearman’s rank correlation coefficient (*r*_*s*_) in at least one of the three forefield systems, showing an increase in the phyla relative abundance going farther away from the glacier toe, whereas Ascomycota, candidate division AD3, and Firmicutes showed both positive and negative *r*_*s*_ in different proglacial systems. The phyla Armatimonadetes, Bacteroidetes, Cyanobacteria, and Firmicutes showed a high abundance in the ice samples and the sites proximal to the ice edge and then decreased in more distant soil (Figure 5C).

### Gene Trends

The PERMANOVA performed on the gene dataset (Table 3C) explained 26% of the observed variance of the samples across different forefields (*p*-value < 0.05) but only 10% of the variance in the GO dataset (Table 3D). Both values decreased when we looked at the datasets without ice (Supplementary Tables 2C,D). The Mantel test statistic performed between the distance from the ice edge and the entire dataset showed a correlation of 0.20. The gene dataset was then significant (*p*-value < 0.05) for SV and SW when correlated to the distance from the ice edge, with *r* equal to 0.47 and 0.37, respectively. In G, the geochemical datasets (TN and TOC) correlated with an *r* of 0.29 (Table 3G).

The 2,422 GO categories found in the proglacial dataset were then checked for statistical correlations with the distances from the ice edge, TN, and TOC. Out of a total of more than 2,400 GO categories, 431 and 110 were positively and negatively correlated to distance, 233 and 374 to TN, and 202 and 328 to TOC. All the GO classes that showed a significant correlation (*p*-value < 0.05) higher than 0.4 or lower than −0.4 were reported in Supplementary Tables 4–6. For example, the distance from the ice edge had a significant positive correlation with oxalate metabolic processes (*r* = 0.61). Also, genes involved in starvation responses (e.g., cellular response to amino acid starvation) or RNA and DNA repair decreased with the increase of the soil distance from the ice front. Furthermore, more genes indicating photosynthetic metabolism were present in early stages of the succession. Finally, distance, TN and TOC positively correlated with the distribution of genes involved in the response to drug and antimicrobial compounds.

#### Nitrogen Fixation

**FIGURE 6:**
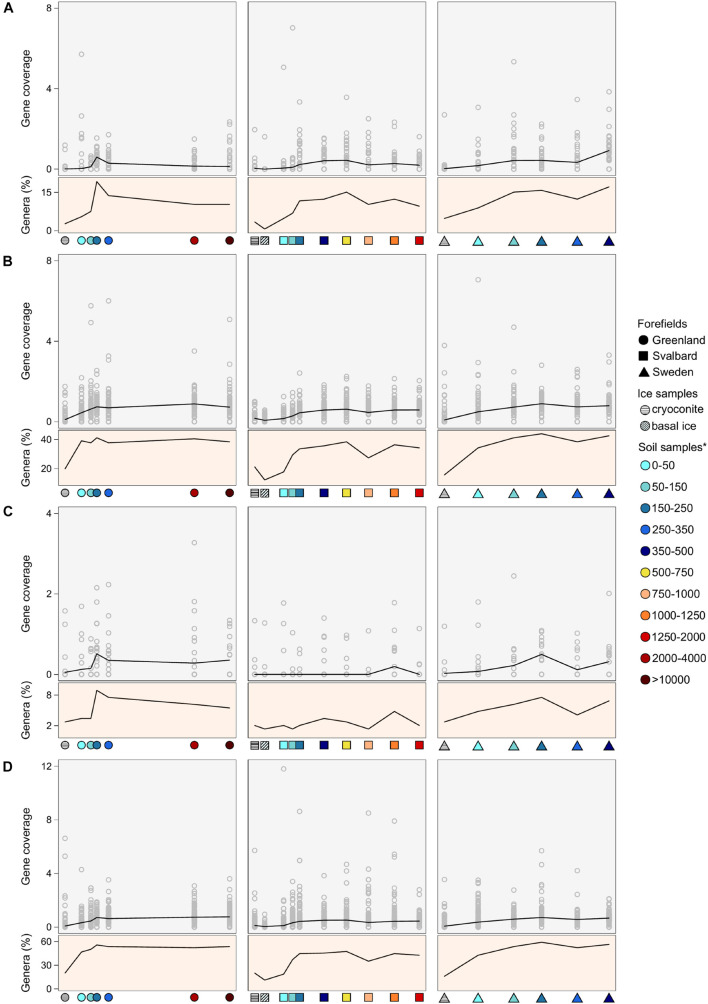
Gene coverage and number of genera trends along the microbial succession for **(A)** nitrogenase genes, **(B)** *obcA* genes that are involved in oxalate biosynthesis, **(C)** cyanide synthase genes, and **(D)** siderophore-related genes. The percentage of genera is calculated as the number of genera that possess the studied gene divided by the total number of genera at a certain distance. *Soil samples are categorized in relation to their distance from the ice edge (m).

No common and clear trends of the nitrogenase gene coverages were observed across the three forefields, where the coverage values peaked at different forefield stages. In the G forefield, the highest coverage of nitrogenase genes was observed at 150–250 m distance, SV showed higher coverage values in the medium soil stages (i.e., 250–750 m), and SW showed a gradual coverage increase with the site distance from the ice edge. The number of genera associated with nitrogenase genes followed the coverage trends (Figure 6A). Whereas Spearman’s correlation coefficient for gene coverage vs. distance was not significant for any of the datasets, it was significant (*p*-value < 0.05) for the G, SV, and SW datasets when performed against the TN data (*r* equals 0.50, 0.30, and 0.54, respectively).

**FIGURE 7:**
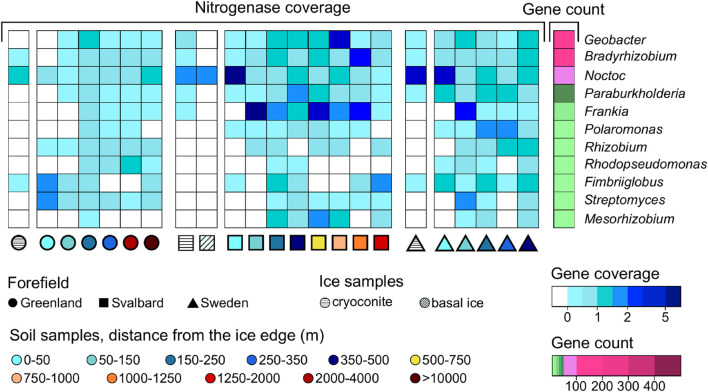
Nitrogenase gene trends at the genus level along the microbial succession. Only genera with more than one coding region associated with a nitrogenase are reported.

Thirty genera were found to have at least one assigned region of the assembly with a nitrogenase coding region. The distance where the majority of taxa had a peak in the nitrogenase coverage was 150–250 m in the G succession, 500–750 m in the SV, and 50–150 m in the SW datasets. *Geobacter*, *Bradyrhizobium*, *Nostoc*, and *Paraburkholderia* had the highest number of genes associated with nitrogenase activity, 156, 101, 53, and 49, respectively (Figure 7). *Nostoc* was the most abundant genus in the ice samples and in the samples closer to the glacier edge, whereas the other three genera showed similar coverages across the soil succession. *Frankia* had 17 contig regions associated with nitrogenase and showed the highest coverage in the early and medium soil stages of the SV and SW soil successions, but it was not present in the ice samples. The other nitrogen-fixing organisms identified from our pipeline were *Polaromonas*, *Rhizobium*, *Rhodopseudomonas*, *Fimbriiglobus*, *Streptomyces*, and *Mesorhizobium* that showed different trends along the soil succession.

Two hundred two assembled coding regions associated with nitrogenases were also associated with unclassified contigs at the genus level. Whereas 50% of the genes were assigned to unknown organisms at the phylum level, 40% of these nitrogenase coding regions were assigned to Proteobacteria and another 5% to Verrucomicrobia (Supplementary Figure 2A).

#### Rock Weathering

**FIGURE 8:**
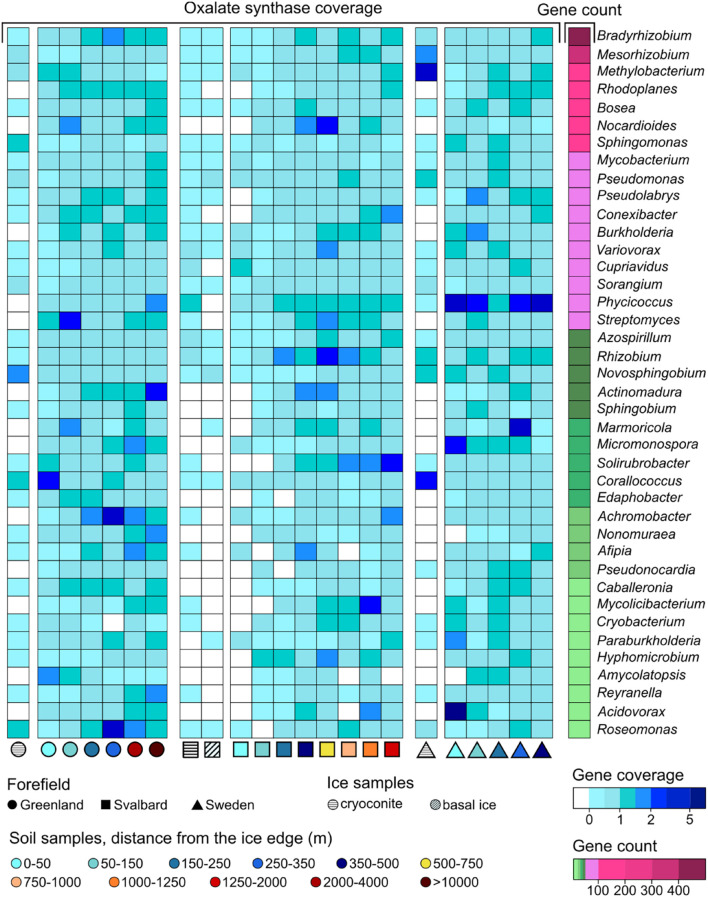
*obcA* gene (involved in oxalate biosynthesis) trends at the genus level along the microbial successions. Only genera with more than 10 coding regions associated with *obcA* genes are reported.

**FIGURE 9:**
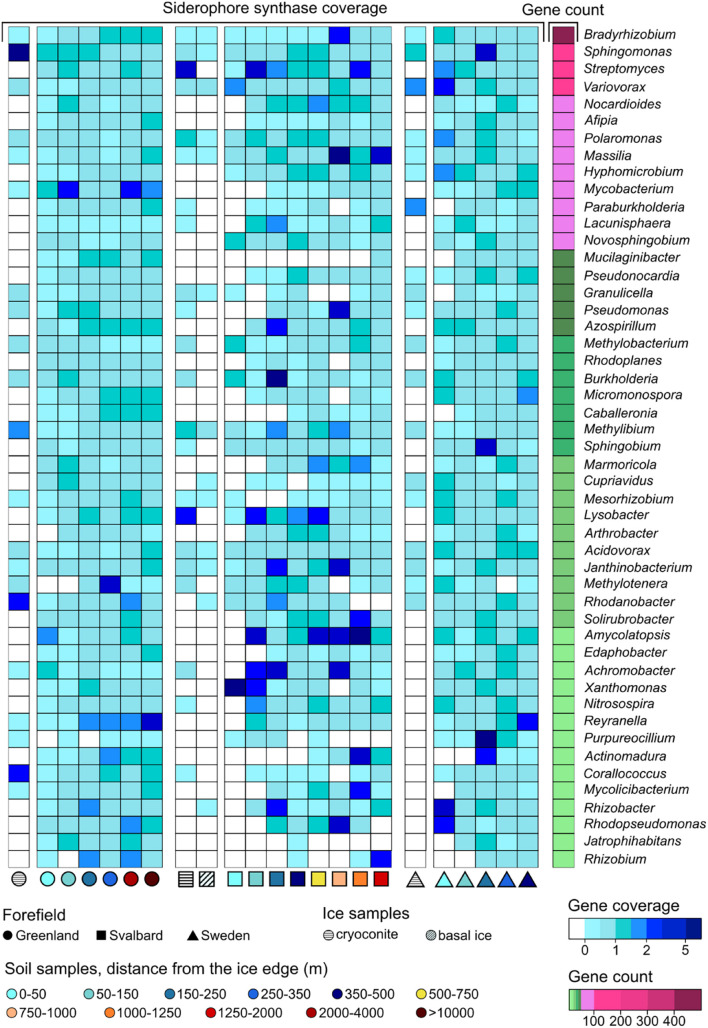
Siderophore-related gene trends at the genus level along the microbial successions. Only genera with more than 10 coding regions associated with siderophore-related genes are reported.

Genes related to rock weathering processes (i.e., *obcA*, cyanide synthase, and genes involved in siderophore synthesis and transport) showed a general lower coverage in the ice and early soil samples and an increase with microbial succession. The number of genera containing these genes followed the coverage trends, increasing with the distance from the ice edge (Figures 6B–D). When looking at the gene distribution at the genus level (Figures 8, 9 and Supplementary Figure 3), we did not observe a common trend of gene distribution. For all the three rock weathering genes, different taxa showed different trends in the same forefield and the same taxon showed different trends in different forefields.

Sixty-five genera had at least one *obcA* gene assigned to their contigs. The genera *Bradyrhizobium*, *Mesorhizobium*, *Methylobacterium*, *Rhodoplanes*, *Bosea*, *Nocardiales*, and *Sphingomonas* had the highest content with 456, 320, 157, 127, 117, 117, and 105 genes, respectively (Figure 8). Some of the other genera showed fewer associated contig areas (i.e., fewer genes), but a high coverage of this gene was involved in oxalate biosynthesis (i.e., *obcA*) at different soil stages. Cyanide synthase genes were less abundant in the three datasets, and only 16 genera were associated with these. The genus *Gemmata* had most of the genes with 18, followed by *Microbacterium* (7) and *Singulisphaera* (5). These genera were also the most ubiquitous in the successions compared to the other cyanide synthase-related genera (Supplementary Figure 3). The three genera that, in particular, showed a higher base coverage in some sites of the microbial succession stages were *Streptomyces*, *Pseudomonas*, and *Variovorax*. More genera were associated with genes involved in siderophore synthesis and transport (81). *Bradyrhizobium*, *Sphingomonas*, *Streptomyces*, and *Variovorax* had most of the genes, and they had overall a higher abundance in the early stages of the SV and SW successions (Figure 9).

The *obcA* gene was also associated with 2,234 unclassified contigs at the genus level, the cyanide synthase gene to 49, and the siderophore genes to 1,919 contig regions. These unclassified contigs mainly belonged to the phyla Proteobacteria, Acidobacteria, Actinobacteria, and Planctomycetes (Supplementary Figures 2B–D).

## Discussion

Microbial successions have been widely studied with the aim of understanding microbial-driven environmental processes and how communities are shaped by environmental factors (Fierer et al., 2010). Glacial forefields constitute ideal systems to study microbial successions, as they are characterized by different environmental gradients, such as an increase in TOC, TN, and vegetation coverage and a decrease in pH; these vary in different soil genesis stages from the bedrocks close to the ice edge to the more developed soil. Many physiochemical factors (e.g., pH, TOC, TN, and water availability) have been shown to influence and shape the microbial diversity and structure at biogeographic scales (Malard and Pearce, 2018) and in forefield specifically (Bradley et al., 2014, 2016; Wang et al., 2020). In our study, we report only two environmental variables (i.e., TOC and TN). Two main biological-driven processes that help create forefield geochemical and microbial trends are nitrogen fixation and rock weathering. We investigated microbial successions from three different proglacial systems with the aims of exploring glacial forefield microbial trends, investigating nitrogen-fixing and rock weathering processes, and exploring the taxonomy associated with these processes.

### Biogeographical Patterns

Whereas the Svalbard and Sweden communities were mainly shaped by their distance from the ice edge, the Greenland communities were more shaped by TN and TOC gradients (Table 3F). This could be due to the nature of its complex dynamics where the GrIS does not retreat linearly and it retreats slower compared to the other systems, leading to more complex dynamics for microbial development (Nienow et al., 2017). The same result was observed when looking at the functional dataset (Table 3G). However, this dataset (not divided into different forefields) also showed a significant correlation with the distance from the ice edge, suggesting that, in this case, certain functional profiles such as photosynthesis and response to starvation or to antimicrobial compounds were conserved across the different forefield gradients (Supplementary Table 4). The variance observed in the gene dataset was also better explained by the forefield differentiation compared to the taxon dataset (Table 3). This indicates how gene and trait-based data could be used in biogeographic studies instead of the more widely used taxonomy data (Green et al., 2008).

### Microbial Taxonomic Succession

In this study, along the microbial succession, microbial communities increased in diversity between ice and soil samples and also with soil complexity (i.e., soil age since deglaciation), where the biggest increase was after the early soil stages of the succession (Figure 3). This was previously observed in Jiang et al. (2018) where bacterial diversity was higher in later soil stages compared to early stages. The first succession stages are characterized by challenging environmental conditions (e.g., nutrient depletion and hydrological disturbances) and are deeply influenced by the glacier environment (characterized by a lower microbial diversity). For these reasons, this soil has a lower community diversity compared to the more developed soil where there is the formation of differentiated ecological niches due to the nutrient increase and plant development (Reynolds et al., 2003; Dumbrell et al., 2010).

In all three proglacial systems, the ice microbial communities comprised of organisms typically found in the cryosphere (Figure 5C). Cyanobacteria, Firmicutes, Bacteroidetes, and Armatimonadetes showed a high abundance in the first stages of the succession where the soil is highly affected by glacial water discharges (Boyd et al., 2014; Bradley et al., 2014; Dieser et al., 2014) and environmental characteristics favor the development of communities adapted to nutrient-depleted conditions. Cyanobacteria and Firmicutes (spore-forming organisms) are widely found in the glacial environment (Galperin, 2016; Segawa et al., 2017). Organisms belonging to the Bacteroidetes phylum have been found in many glacial habitats (e.g., Zhang et al., 2009; Wilhelm et al., 2013; Smith et al., 2016). The diffused presence of these non-spore-forming heterotrophic organisms has been proposed to be related to their ability to assimilate and use recalcitrant substances (Zeng et al., 2013) abundant in the glacier environment (Kmezik et al., 2020). Organisms belonging to Armatimonadetes have been found in this environment (e.g., Bajerski and Wagner, 2013; Zhang et al., 2015; Gokul et al., 2016). This phylum is not well characterized, but its few isolates have been characterized as chemoheterotrophs (Tamaki et al., 2011; Lee et al., 2014).

Whereas in the first soil stages of the succession, microbial diversity showed a presence of autotrophic organisms (i.e., Cyanobacteria), spore-forming organisms (i.e., Firmicutes), and organisms specialized to use recalcitrant compounds (i.e., Bacteroidetes), processes and organisms associated with heterotrophy were shown to increase in later stages of the microbial succession. In the three proglacial systems, microbial succession was characterized by the increase of Acidobacteria (Figure 5C). Organisms belonging to this phylum are heterotrophs and adapted to use a variety of organic carbon sources. Furthermore, many of these organisms are acidophiles (Kielak et al., 2016) and therefore more adapted to live in the later stages of the succession where the soil is enriched of organic carbon and pH is lower (Zumsteg et al., 2012). Other phyla such as *Candidatus* Rokubacteria, Elusimicrobia, Nitrospirae, and Planctomycetes showed an increased abundance in the more developed soil of the later stages. Elusimicrobia have been isolated mainly from soil and are principally insect symbionts (Méheust et al., 2019). *Candidatus* Rokubacteria and Verrucomicrobia are characterized by organisms able to perform sulfate reduction (Anantharaman et al., 2018). Nitrospirae metabolism relies on nitrite oxidation (Daims et al., 2016). These microorganisms are favored in the later stages of the succession where nitrogen and sulfur stocks have been built by diazotrophs and rock weathering organisms. Actinobacteria and Proteobacteria (Alphaproteobacteria in particular) showed oscillating trends along the three proglacial environments (Figure 5A and Supplementary Table 3). Actinobacteria and Proteobacteria are two ubiquitous phyla and are both characterized by a wide metabolic range (Barka et al., 2016; Taylor et al., 2019). However, a change in the relative abundance of these two phyla has previously been observed in relation to different soil matrices and mineralogy that can be encountered both at small-scale distances and along forefield successions in soil (Mitchell et al., 2013; Uroz et al., 2015).

In the soil successions, the main CO_2_-fixing organisms in the early successional soil stages belonged to the phylum Cyanobacteria, where *Nostoc* was the most abundant genus. In later soil stages, autotrophic organisms were ascribed to several microbial phyla, such as Planctomycetes, Nitrospirae, Chloroflexi, Proteobacteria, and Verrucomicrobia (Xu et al., 2017; Chen et al., 2018; Li et al., 2019). Most of these phyla showed an increase along the soil successions, in particular in the SV and SW forefields where a more linear soil chronosequence was present (Figures 1, 5C). This suggests phototrophs to be substituted by chemolithoautotrophic organisms along forefield microbial successions, where more nutrients are present in later soil stages to sustain chemolithoautotrophic metabolisms.

Although ice-related/soil-related organism trends were observed in all the proglacial systems, the latter showed differences in relative abundances of several taxa (e.g., Nitrospirae, *Candidatus* division AD3, Chloroflexi, and Gammatimonadetes), showing how local patterns and characteristics could influence the microbial distribution and the biogeography of different soil microbial communities. Environmental selection to specific conditions (e.g., DOC, TN, and pH) has previously been observed to strongly shape microbial communities at large biogeographic spatial scales (Hanson et al., 2012; Malard and Pearce, 2018). Highlighting this aspect, no correlation between distance from the ice edge and the entire forefield dataset was observed; a high correlation was obtained only when looking at the microbial distribution across the separate forefields (Table 3F), showing how the forefield local factors had a role in shaping the microbial communities.

### Functional Succession

We investigated the taxonomy of the organisms performing two important microbially driven environmental processes: nitrogen fixation and rock weathering. To do so, we focused on four different sets of genes: (i) nitrogenase genes, (ii) *obcA* genes involved in the first step of the oxalate biosynthesis, (iii) genes involved in cyanide synthesis, and (iv) genes involved in siderophore synthesis and transport. Whereas the first one is involved in nitrogen fixation, the other three are involved in rock weathering processes where oxalate and cyanide have been observed to increase rock and mineral dissolution and siderophore is a molecule used to facilitate iron uptake inside the cells. These processes have previously been shown to be prevalent closer to the ice edge where microorganisms that are able to produce metabolic energy from atmospheric nitrogen and inorganic compounds (e.g., minerals) are favored (Brankatschk et al., 2011; Fernández-Martínez et al., 2016).

In all three systems, genes associated with these processes were shared by the lowest percentage of taxa in the early stages and by more in later succession stages (Figures 6–9). Interestingly, nitrogenase genes showed a correlation with TN concentration values, highlighting the pivotal role of diazotrophs in the creation of a nitrogen pool in proglacial systems (Nash et al., 2018). Gene coverages observed at specific genus levels showed a variety of trends and distributions, suggesting that different organisms drive the same ecological processes (i.e., nitrogen fixation and rock weathering) but at different stages of microbial succession.

#### Taxa Involved in Nitrogen Fixation

Nitrogenases are key enzymes in the fixation of atmospheric nitrogen to ammonium. These enzymes are coded by a wide gene class (e.g., *nifK*, *nifH*, and *nifD* genes) and have been widely used to detect biological nitrogen fixation potential in a variety of environmental settings (Hoffman et al., 2014). Further, diazotrophic organisms have been found spanning a wide range of different bacterial phyla (Addo and Dos Santos, 2020).

Most of the 30 genera associated with nitrogenase genes in our dataset belonged to the phyla Proteobacteria (17) and Actinobacteria (6). The only cyanobacterial organism to which nitrogenase was assigned in our dataset was *Nostoc*, which also showed a higher abundance in the ice realm compared to the proglacial soil and has previously been detected by Fernandez et al. (2020) in the Arctic region. The other cyanobacterial organisms present in our dataset were present in a lower percentage (1%) compared to *Nostoc* (4%); between them, there are, for example, *Phormidesmis* and *Chamaesiphon*. These organisms are typical of the cold environments and do not perform nitrogen fixation (Jungblut and Vincent, 2017; Segawa et al., 2017; Uetake et al., 2019). In the soil, diazotrophic organisms belonging to Proteobacteria and Actinobacteria were more abundant (Figure 7). These phyla are usually associated with soil environment and root symbiosis (Rudnick et al., 1997; Gtari et al., 2012). In addition to *Nostoc*, the nitrogen fixers *Bradyrhizobium*, *Geobacter*, *Paraburkholderia*, and *Frankia* (Sellstedt and Richau, 2013; Calderoli et al., 2017; Thangaraj et al., 2017; Choi and Im, 2018; Hara et al., 2019) were abundant in all our studied systems (Figure 7). The same dominant nitrogen fixers were found in Nash et al. (2018) where *Nostoc*, *Geobacter*, *Rhizobium*, *Polaromonas*, and *Frankia* were found dominant in all three forefield systems. In our study, *Streptomyces* was also found associated with nitrogenase genes. This genus has only recently been shown to perform nitrogen fixation by isolation and sequencing from environmental soil (Dahal et al., 2017). This result represents the first confirmation of the presence of nitrogenase genes in *Streptomyces*. Furthermore, the genus *Fimbriiglobus* was also associated with these genes in our dataset. The only organism previously sequenced belonging to this genus (*Fimbriiglobus ruber*) does not have nitrogen-fixing genes (Ravin et al., 2018). However, our result could suggest that other members of the genus do.

#### Taxa Involved in Rock Weathering

Compared to biological nitrogen fixation, less is known about biological rock weathering. Whereas some microbial processes involved in the rock weathering have already been identified, the protagonists of these processes still remain unclear (Samuels et al., 2020). We explored the taxonomy associated with three different rock weathering-associated genes: the *obcA* gene, cyanide synthase genes, and genes involved in siderophore transport and synthesis. Whereas oxalate, cyanide, and siderophore syntheses have been shown to be correlated with rock weathering (Welch et al., 1999; Frey et al., 2010; Ferreira et al., 2019), these three molecules are involved in other processes. For instance, the release of oxalate has been shown to play an important role in pathogenicity and metal tolerance (Palmieri et al., 2019) and cyanide release plays a role in microbial competition (Blumer and Haas, 2000). Siderophores are molecules commonly used by microorganisms for the uptake of environmental iron but also to sequester iron from hosts (Krewulak and Vogel, 2008; Braun and Hantke, 2011). Even if all of these processes are present in organisms not involved in rock weathering, we expect a higher abundance of these organisms in early succession samples where the rock weathering is predominant. However, the genera associated with rock-weathering genes did not show common and continuous trends along the chronosequence, suggesting that different organisms can conduct a certain environmental function (e.g., rock dissolution) at different succession stages and soil conditions (Figures 8, 9 and Supplementary Figure 3).

The genus that showed most of the genes involved in oxalate and siderophore biosynthesis was *Bradyrhizobium*. Organisms belonging to this genus are mainly recognized as plant symbionts but can also be present as free-living organisms (Kulkarni et al., 2015; Čuklina et al., 2016). The high content and diversity of genes involved in siderophore metabolism relate to the fact that high production of siderophore is needed to uptake iron released from the rock dissolution performed by oxalate release. These organisms are also diazotrophs and require iron as a nitrogenase cofactor (Rubio and Ludden, 2008). *Mesorhizobium* and *Paraburkholderia* are also common diazotrophic components of the rhizosphere but have been detected as free-living organisms (Ahmad et al., 2008), and they showed high presence of both these genes. *Paraburkholderia* was found to have chemotactic sensitivity for oxalate even if it was not directly shown that it can produce it (Haq et al., 2018). *Micromonospora* also had high oxalate-related gene abundance especially in the first stages of the SW dataset. Organisms related to this genus have been found in soil by different studies (Thawai et al., 2016; Malisorn et al., 2020) and have previously been found to increase phosphate and iron solubilization in soil due to organic acid release, such as oxalate (El-Tarabily et al., 2008). Overall, *Burkholderia* had a higher presence of both siderophore and oxalate-related genes in early stages compared to later soil stages. These organisms have been identified by many different studies as oxalate producers (Nakata, 2011; Oh et al., 2014) and often have been found in proglacial soil (Frey et al., 2010). *Pseudomonas* was shown to produce oxalic acids (Hamel et al., 1999), which concords with the trends in our datasets. Additionally, contigs associated with *Pseudomonas* were found to contain cyanide synthase-coding regions. *Streptomyces* was previously identified as a phosphate solubilizer, but mechanisms were not identified (Liu et al., 2016). Other than the oxalate genes, this genus was also shown to have cyanide synthase genes in our dataset. Finally, *Fimbriiglobus*, *Corallococcus*, *Variovorax*, and *Phycicoccus* are organisms typically found in soil (Yoon et al., 2008; Zhang et al., 2011; Singh et al., 2015) and were associated with oxalate, cyanide, and siderophore genes for the first time in this study.

## Conclusion

Microbial successions in the three proglacial systems showed early soil stages enriched with autotrophic, spore-forming, and recalcitrant compound degraders showing a community influenced by the common ice microbiome, whereas later soil stages showed a higher heterotrophic microbial component. Although there were common taxonomic trends among the proglacial systems, taxon contribution to the different proglacial microbial communities showed differences, suggesting the presence of biogeographic differences between proglacial systems. Furthermore, rock weathering and nitrogenase gene distributions peaked at different succession soil stages in different proglacial systems. Different genera showed different gene coverages at different stages of the microbial successions, indicating a community constituted by a plurality of organisms involved in these processes, and where the latter were driven by different organisms in different soil succession stages. Whereas we confirmed the presence of nitrogenase and rock weathering genes in known nitrogen-fixing and rock weathering organisms, in this study, we also present organisms that, even if often found in soil and proglacial systems, have never been related to these processes before. These results represent a further step toward a more comprehensive understanding of nitrogen-fixing and biotic rock weathering processes and highlight how a better understanding of these and other microbially driven environmental processes (e.g., CO_2_ fixation) is necessary for a better comprehension of microbial successions in a warming Arctic.

## Data Availability Statement

Publicly available datasets were analyzed in this study. This data can be found here: https://www.ebi.ac.uk/ena/browser/view/PRJEB41174.

## Author Contributions

All authors wrote the manuscript. GV analyzed the data and performed the statistical analysis.

## Conflict of Interest

The authors declare that the research was conducted in the absence of any commercial or financial relationships that could be construed as a potential conflict of interest. The handling editor declared a past co-authorship with one of the authors GB.

## Publisher’s Note

All claims expressed in this article are solely those of the authors and do not necessarily represent those of their affiliated organizations, or those of the publisher, the editors and the reviewers. Any product that may be evaluated in this article, or claim that may be made by its manufacturer, is not guaranteed or endorsed by the publisher.

## Footnotes

1
https://www.bioinformatics.babraham.ac.uk/projects/fastqc/

2
https://github.com/gvMicroarctic/LongMeta
