# Supplementary material for: A Taxon-Wise Insight Into Rock Weathering and Nitrogen Fixation Functional Profiles of Proglacial Systems
Source: Front Microbiol. 2021 Sep 16;12:627437. doi: 10.3389/fmicb.2021.627437 (PMC8491546; doi:10.3389/fmicb.2021.627437)
Supplement: Supplementary file 1 [file Data_Sheet_1.docx]

Supplementary Material

**A taxon-wise insight into rock weathering and nitrogen fixation functional profiles of proglacial systems**

**Gilda Varliero, Alexandre M. Anesio, Gary L. A. Barker**

**Correspondence: Gilda Varliero** (gilda.varliero@bristol.ac.uk)

# List Supplementary Materials:

**Figure S1.** Distance-based redundancy analysis (dbRDA) bi-plot ordination performed on the Hellinger-transformed genus dataset in relation to the distance from the glacier toe, TOC and TN. Only genera that had a dbRDA1 or dbRDA2 higher than 0.15 or lower than -0.15 are displayed in the plot. Vectors indicate direction of the ice edge distance and geochemical variable effect in the bacterial community composition (Bray-Curtis similarity).

**Figure S2.** Phyla related to the unclassified coding region at the genus-level for nitrogenase genes **(A)**, *obcA* genes which are involved in the oxalate biosynthesis **(B)**, cyanide synthase genes **(C)** and siderophore-related genes **(D)**.

**Figure S3.** Cyanide synthase genes at genus-level along the microbial successions.

**Table S1.** Read content per sample. All the counts are reported as the sum of both forward and reverse reads. Reads from G and SW datasets were 150 bp long, whereas those from SV dataset were 100 bp long.

**Table S2.** Statistical analyses performed on the entire dataset (soil + ice samples). Permutational multivariate analysis of variance (PERMANOVA) performed between the distance from the ice edge and the diversity index dataset **(A)**, taxonomy dataset at the phylum-, order- and genus- level (B), gene dataset **(C)** and the GO (Gene Ontology) dataset **(D)**. Mantel test performed to calculate the correlation between the distance from the ice edge or the geochemical dataset (i.e. TN + TOC) with the diversity index dataset **(E)**, taxonomy dataset at the phylum-, order- and genus- level (F), gene dataset **(G)** and the GO (Gene Ontology) dataset (H). Each of these four datasets were tested with all the samples from the three different proglacial systems (G + SV + SW), only the G system, only the SV system and only the SW system. The symbol '*' is reported for significant significant *R*^2^ and *r* values where the statistic *p*-value < 0.05.

**Table S3.** Actinobacteria and Proteobacteria class abundance linear model correlation (*R^2^*). The symbol `-' is reported when the correlation was not significant with a *p*-value ≥ 0.05. *Actinobacteria classes. **Proteobacteria classes.

**Table S4.** Gene Ontology (GO) biological categories that significantly correlated (Mantel test statistic, *p*-value < 0.05) to the distances from the ice edge.

**Table S5.** Gene Ontology (GO) biological categories that significantly correlated (Mantel test statistic, *p*-value < 0.05) to the total organic carbon distribution (TOC).

**Table S6.** Gene Ontology (GO) biological categories that significantly correlated (Mantel test statistic, *p*-value < 0.05) to the total nitrogen distribution (TN).

Figure S1. Distance-based redundancy analysis (dbRDA) bi-plot ordination performed on the Hellinger-transformed genus dataset in relation to the distance from the glacier toe, TOC and TN. Only genera that had a dbRDA1 or dbRDA2 higher than 0.15 or lower than -0.15 are displayed in the plot. Vectors indicate direction of the ice edge distance and geochemical variable effect in the bacterial community composition (Bray-Curtis similarity).


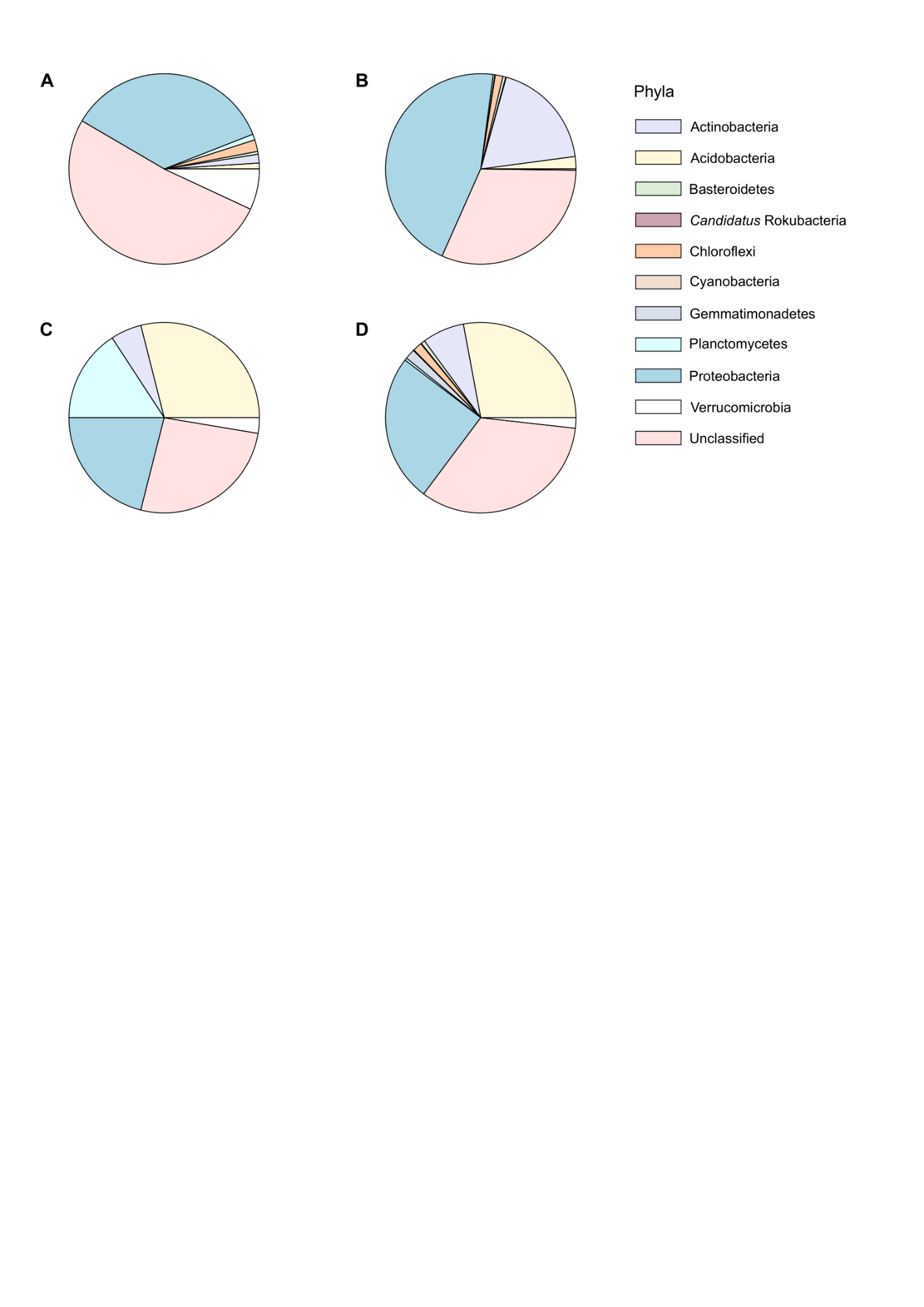


Figure S2. Phyla related to the unclassified coding region at the genus-level for nitrogenase genes (A), *obcA* genes which are involved in the oxalate biosynthesis (B), cyanide synthase genes (C) and siderophore-related genes (D).


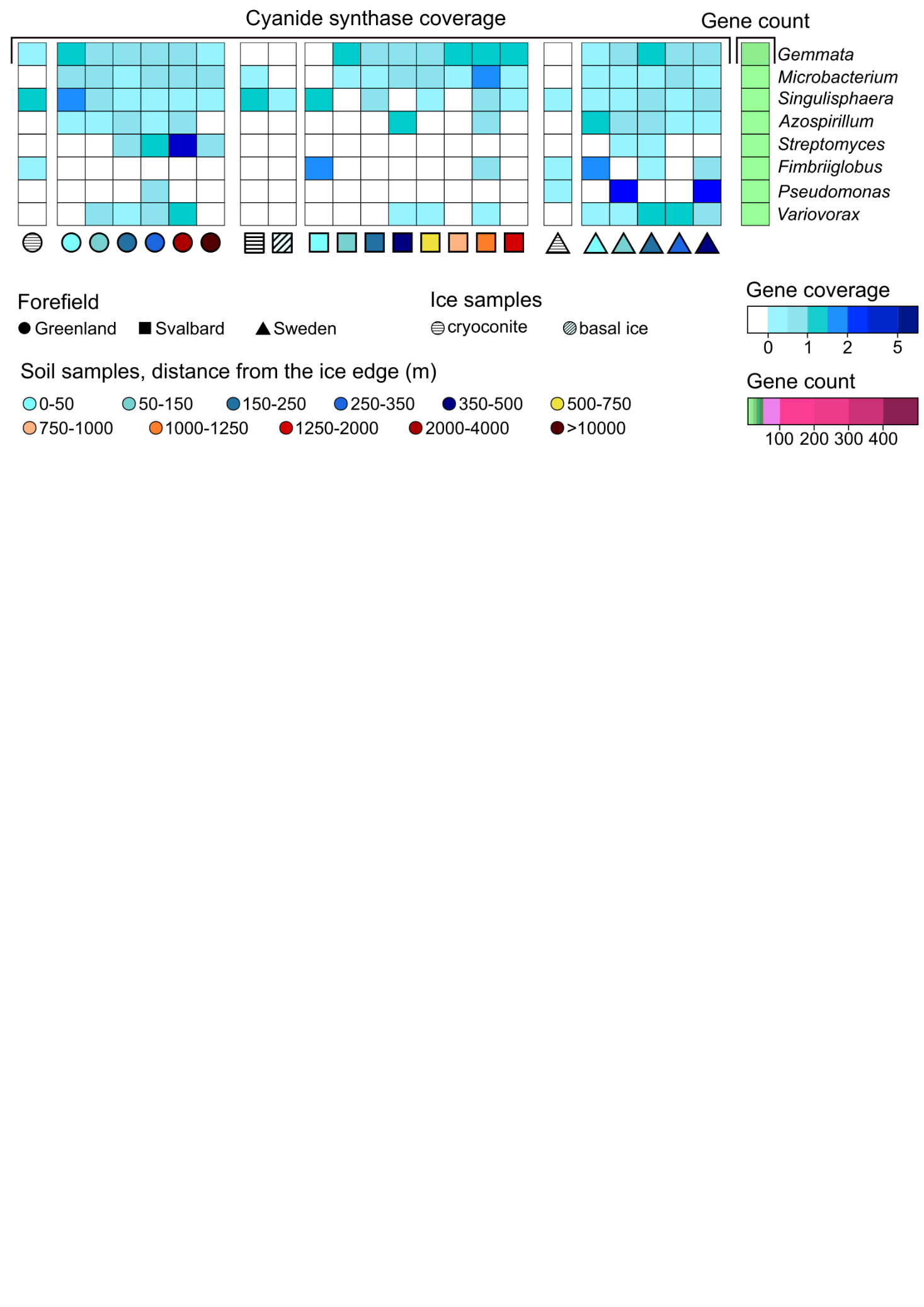


Figure S3. Cyanide synthase genes at genus-level along the microbial successions.

Table S1. Read content per sample. All the counts are reported as the sum of both forward and reverse reads. Reads from G and SW datasets were 150 bp long, whereas those from SV dataset were 100 bp long.

| **Sample ID** | **Forefield** | **Distance** | **Raw reads** | **Trimmed reads** | **Trimmed reads (%)** | **Total bases** | **Mapped reads to assembly (%)** |
| --- | --- | --- | --- | --- | --- | --- | --- |
| G-1 | G | cryoconite | 74,992,750 | 62,281,172 | 83 | 7,187,824,916 | 94 |
| G-2 | G | cryoconite | 74,220,220 | 62,697,712 | 84 | 7,395,859,056 | 94 |
| G-3 | G | cryoconite | 68,161,082 | 56,551,528 | 83 | 6,528,966,072 | 93 |
| G-4 | G | 40 | 67,748,804 | 58,269,730 | 86 | 6,964,519,188 | 69 |
| G-5 | G | 40 | 77,474,456 | 66,716,324 | 86 | 7,985,703,488 | 68 |
| G-6 | G | 40 | 61,199,512 | 50,778,584 | 83 | 5,731,546,733 | 62 |
| G-7 | G | 120 | 75,975,046 | 64,894,402 | 85 | 7,687,561,070 | 72 |
| G-8 | G | 120 | 91,844,214 | 80,750,866 | 88 | 9,918,813,542 | 78 |
| G-9 | G | 120 | 77,023,868 | 66,570,850 | 86 | 8,011,592,250 | 71 |
| G-10 | G | 160 | 139,381,778 | 119,421,182 | 86 | 14,179,337,314 | 59 |
| G-11 | G | 160 | 91,962,344 | 77,483,224 | 84 | 9,026,364,457 | 56 |
| G-12 | G | 160 | 81,681,122 | 70,366,000 | 86 | 8,417,841,387 | 58 |
| G-13 | G | 180 | 105,431,412 | 89,380,892 | 85 | 10,505,678,835 | 65 |
| G-14 | G | 180 | 63,240,678 | 53,430,508 | 84 | 6,276,219,348 | 68 |
| G-15 | G | 180 | 72,243,790 | 60,669,410 | 84 | 7,127,227,196 | 63 |
| G-16 | G | 270 | 82,137,112 | 71,117,502 | 87 | 8,597,039,587 | 64 |
| G-17 | G | 270 | 107,570,706 | 90,819,662 | 84 | 10,578,631,872 | 65 |
| G-18 | G | 270 | 78,274,268 | 66,493,958 | 85 | 7,850,171,960 | 65 |
| G-19 | G | 3800 | 75,392,190 | 63,919,782 | 85 | 7,552,035,227 | 57 |
| G-20 | G | 3800 | 65,582,044 | 55,780,594 | 85 | 6,587,075,487 | 50 |
| G-21 | G | 3800 | 91,522,052 | 76,517,016 | 84 | 8,858,708,214 | 64 |
| G-22 | G | 10000 | 96,384,726 | 81,771,096 | 85 | 9,655,404,024 | 62 |
| G-23 | G | 10000 | 80,981,820 | 68,533,676 | 85 | 7,984,763,989 | 59 |
| SV-1 | SV | cryoconite | 22,947,810 | 22,269,794 | 97 | 2,215,189,703 | 75 |
| SV-2 | SV | basal ice | 28,894,400 | 28,395,640 | 98 | 2,817,953,846 | 93 |
| SV-3 | SV | 40 | 18,800,440 | 17,537,922 | 93 | 1,742,261,993 | 64 |
| SV-4 | SV | 130 | 28,886,708 | 28,303,382 | 98 | 2,813,720,107 | 52 |
| SV-5 | SV | 130 | 18,972,122 | 16,906,176 | 89 | 1,676,259,798 | 56 |
| SV-6 | SV | 200 | 27,632,502 | 25,897,482 | 94 | 2,576,342,099 | 46 |
| SV-7 | SV | 200 | 23,060,890 | 20,724,024 | 90 | 2,059,258,461 | 44 |
| SV-8 | SV | 200 | 22,002,334 | 21,530,160 | 98 | 2,137,672,181 | 48 |
| SV-9 | SV | 445 | 23,356,976 | 22,759,102 | 97 | 2,260,867,633 | 48 |
| SV-10 | SV | 445 | 20,355,874 | 20,087,560 | 99 | 1,996,581,694 | 52 |
| SV-11 | SV | 445 | 23,227,446 | 22,764,896 | 98 | 2,260,324,489 | 49 |
| SV-12 | SV | 670 | 59,467,440 | 58,657,474 | 99 | 5,826,895,594 | 48 |
| SV-13 | SV | 670 | 57,473,614 | 56,788,254 | 99 | 5,640,225,267 | 47 |
| SV-14 | SV | 670 | 38,289,390 | 38,032,472 | 99 | 3,786,740,956 | 47 |
| SV-15 | SV | 890 | 4,048,326 | 3,967,012 | 98 | 394,610,874 | 32 |
| SV-16 | SV | 890 | 22,892,266 | 22,324,698 | 98 | 2,213,591,398 | 40 |
| SV-17 | SV | 890 | 8,664,656 | 6,543,310 | 76 | 643,999,833 | 34 |
| SV-18 | SV | 1150 | 22,630,114 | 22,343,416 | 99 | 2,217,910,996 | 45 |
| SV-19 | SV | 1150 | 39,315,396 | 38,856,178 | 99 | 3,861,257,149 | 42 |
| SV-20 | SV | 1150 | 69,568,490 | 68,761,830 | 99 | 6,822,952,060 | 44 |
| SV-21 | SV | 1650 | 24,043,000 | 23,513,418 | 98 | 2,335,643,616 | 43 |
| SV-22 | SV | 1650 | 20,762,772 | 20,273,822 | 98 | 2,010,967,936 | 51 |
| SV-23 | SV | 1650 | 29,280,810 | 28,867,114 | 99 | 2,874,021,170 | 47 |
| SW-1 | SW | cryoconite | 62,395,094 | 59,053,216 | 95 | 8,220,938,496 | 92 |
| SW-2 | SW | 40 | 78,397,352 | 74,222,178 | 95 | 10,443,960,493 | 79 |
| SW-3 | SW | 130 | 66,910,678 | 63,704,550 | 95 | 8,951,219,174 | 78 |
| SW-4 | SW | 130 | 72,683,122 | 69,228,276 | 95 | 9,719,225,957 | 77 |
| SW-5 | SW | 130 | 60,148,730 | 57,046,786 | 95 | 7,892,712,713 | 75 |
| SW-6 | SW | 130 | 67,844,804 | 64,231,264 | 95 | 8,923,085,594 | 72 |
| SW-7 | SW | 130 | 63,953,088 | 60,944,452 | 95 | 8,542,182,607 | 73 |
| SW-8 | SW | 200 | 83,010,234 | 79,354,112 | 96 | 11,161,621,182 | 73 |
| SW-9 | SW | 200 | 74,901,072 | 71,515,238 | 95 | 10,030,231,547 | 72 |
| SW-10 | SW | 200 | 83,405,572 | 79,804,612 | 96 | 11,223,988,377 | 70 |
| SW-11 | SW | 200 | 68,111,048 | 64,777,286 | 95 | 9,100,927,086 | 72 |
| SW-12 | SW | 290 | 64,225,756 | 61,152,466 | 95 | 8,552,527,075 | 66 |
| SW-13 | SW | 290 | 64,764,076 | 61,854,200 | 96 | 8,671,030,908 | 71 |
| SW-14 | SW | 370 | 66,675,200 | 63,459,146 | 95 | 8,874,543,259 | 65 |
| SW-16 | SW | 370 | 66,848,090 | 63,779,024 | 95 | 8,945,037,456 | 71 |
| SW-16 | SW | 370 | 72,051,286 | 68,487,298 | 95 | 9,580,949,996 | 76 |
| SW-17 | SW | 370 | 71,828,498 | 68,123,690 | 95 | 9,552,272,522 | 66 |
| SW-18 | SW | 370 | 85,214,054 | 81,120,886 | 95 | 11,368,376,573 | 71 |
| SW-19 | SW | 370 | 71,411,294 | 68,058,210 | 95 | 9,559,127,942 | 67 |

Table S2. Statistical analyses performed on the entire dataset (soil + ice samples). Permutational multivariate analysis of variance (PERMANOVA) performed between the distance from the ice edge and the diversity index dataset (A), taxonomy dataset at the phylum-, order- and genus- level (B), gene dataset (C) and the GO (Gene Ontology) dataset (D). Mantel test performed to calculate the correlation between the distance from the ice edge (Distance) or the geochemical dataset (i.e. TN + TOC) with the diversity index dataset (E), taxonomy dataset at the phylum-, order- and genus- level (F), gene dataset (G) and the GO (Gene Ontology) dataset (H). Each of these four datasets were tested with all the samples from the three different proglacial systems (G + SV + SW), only the G system, only the SV system and only the SW system. The symbol '*' is reported for significant *R*^2^ and *r* values where the statistic *p*-value < 0.05.

| **A** | **Diversity indices** | |  | **E** | **Diversity indices** | | | | |
| --- | --- | --- | --- | --- | --- | --- | --- | --- | --- |
|  | ***R^2^*** | ***p*-value** |  |  | **Forefield** | **Distance** | | **TN + TOC** | |
|  | 0.12 | 0.01* |  |  |  | ***r*** | ***p*-value** | ***r*** | ***p*-value** |
|  |  |  |  |  | G + SV + SW | 0.02 | 0.35 | -0.07 | 0.86 |
|  |  |  |  |  | G | 0.19 | 0.06 | 0.03 | 0.32 |
|  |  |  |  |  | SV | 0.45 | 0.00* | 0.01 | 0.42 |
|  |  |  |  |  | SW | 0.18 | 0.07 | 0.13 | 0.20 |
|  |  |  |  |  |  |  |  |  |  |
| **B** | **Taxonomy** | | | **F** | **Taxonomy** | | | | |
|  | **Rank** | ***R^2^*** | ***p*-value** |  | **Forefield** | **Distance** | | **TN + TOC** | |
|  | phylum | 0.10 | 0.01* |  |  | ***r*** | ***p*-value** | ***r*** | ***p*-value** |
|  | order | 0.16 | 0.00* |  | G + SV + SW | 0.14 | 0.03* | 0.01 | 0.39 |
|  | genus | 0.15 | 0.00* |  | G | 0.26 | 0.02* | 0.18 | 0.06 |
|  |  |  |  |  | SV | 0.61 | 0.00* | 0.06 | 0.31 |
|  |  |  |  |  | SW | 0.40 | 0.00* | 0.28 | 0.06 |
|  |  |  |  |  |  |  |  |  |  |
| **C** | **Gene** | |  | **G** | **Gene** | | | | |
|  | ***R^2^*** | ***p*-value** |  |  | **Forefield** | **Distance** | | **TN + TOC** | |
|  | 0.21 | 0.00* |  |  |  | ***r*** | ***p*-value** | ***r*** | ***p*-value** |
|  |  |  |  |  | G + SV + SW | 0.27 | 0.00* | -0.02 | 0.59 |
|  |  |  |  |  | G | 0.28 | 0.02* | 0.14 | 0.06 |
|  |  |  |  |  | SV | 0.53 | 0.00* | 0.09 | 0.25 |
|  |  |  |  |  | SW | 0.50 | 0.00* | 0.34 | 0.04* |
|  |  |  |  |  |  |  |  |  |  |
| **D** | **GO categories** | |  | **H** | **GO categories** | | | | |
|  | ***R^2^*** | ***p*-value** |  |  | **Forefield** | **Distance** | | **TN + TOC** | |
|  | 0.08 | 0.00* |  |  |  | ***r*** | ***p*-value** | ***r*** | ***p*-value** |
|  |  |  |  |  | G + SV + SW | 0.09 | 0.12 | -0.13 | 0.92 |
|  |  |  |  |  | G | 0.56 | 0.00* | -0.02 | 0.53 |
|  |  |  |  |  | SV | 0.37 | 0.00* | -0.08 | 0.66 |
|  |  |  |  |  | SW | 0.25 | 0.03* | 0.09 | 0.28 |

Table S3. Actinobacteria and Proteobacteria class abundance linear model correlation (*R^2^*). The symbol `-' is reported when the correlation was not significant with a *p*-value ≥ 0.05. *Actinobacteria classes. **Proteobacteria classes.

|  | **Actinobacteria*** | | **Acidimicrobiia*** | | **Rubrobacteria*** | | **Thermoleophilia*** | |  |
| --- | --- | --- | --- | --- | --- | --- | --- | --- | --- |
|  | ***R^2^*** | ***p*-value** | ***R^2^*** | ***p*-value** | ***R^2^*** | ***p*-value** | ***R^2^*** | ***p*-value** | |
| **Alphaproteobacteria**** | 0.34 | 0.00 | 0.22 | 0.00 | 0.20 | 0.00 | - | - | |
| **Betaproteobacteria**** | 0.02 | 0.26 | 0.08 | 0.02 | - | - | 0.22 | 0.00 | |
| **Gammaproteobacteria**** | 0.20 | 0.00 | 0.20 | 0.00 | 0.13 | 0.00 | 0.23 | 0.00 | |
| **Deltaproteobacteria**** | 0.16 | 0.00 | 0.13 | 0.00 | 0.09 | 0.01 | 0.14 | 0.00 | |

Table S4. Gene Ontology (GO) biological categories that significantly correlated (Mantel test statistic, *p*-value < 0.05) to the distances from the ice edge.

| **Biological functions** | **positive r** |
| --- | --- |
| fumarate transport | 0.65 |
| succinate transmembrane transport | 0.65 |
| purine-containing compound salvage | 0.58 |
| xanthine metabolic process | 0.58 |
| pentose metabolic process | 0.54 |
| tRNA 3'-trailer cleavage, endonucleolytic | 0.52 |
| NADP metabolic process | 0.51 |
| lipoprotein metabolic process | 0.51 |
| response to drug | 0.49 |
| glycerophosphodiester transmembrane transport | 0.49 |
| sodium ion export across plasma membrane | 0.48 |
| potassium ion export across plasma membrane | 0.48 |
| response to osmotic stress | 0.47 |
| cellular monovalent inorganic cation homeostasis | 0.47 |
| histone modification | 0.47 |
| xenobiotic catabolic process | 0.47 |
| regulation of viral transcription | 0.46 |
| toxin metabolic process | 0.46 |
| peptidyl-lysine demalonylation | 0.46 |
| peptidyl-lysine desuccinylation | 0.46 |
| chromate transport | 0.46 |
| tRNA 3'-trailer cleavage | 0.45 |
| branched-chain amino acid catabolic process | 0.45 |
| valine catabolic process | 0.44 |
| D-xylose transmembrane transport | 0.44 |
| amino-acid betaine catabolic process | 0.44 |
| glycerol-3-phosphate transmembrane transport | 0.43 |
| xenobiotic transport | 0.43 |
| alpha-glucan catabolic process | 0.43 |
| cellular oligosaccharide catabolic process | 0.43 |
| alkaloid metabolic process | 0.42 |
| 2,4-dichlorophenoxyacetic acid catabolic process | 0.42 |
| cholesterol biosynthetic process | 0.41 |
| galactitol transport | 0.41 |
| peptide metabolic process | 0.41 |
| L-threonine catabolic process to glycine | 0.41 |
| glucose transmembrane transport | 0.41 |
| D-xylose metabolic process | 0.40 |
| cellular response to acidic pH | 0.40 |
| XMP salvage | 0.40 |
| antibiotic catabolic process | 0.40 |
| alginic acid biosynthetic process | 0.40 |
| leucine catabolic process | 0.40 |
| **Biological functions** | **negative r** |
| tRNA seleno-modification | -0.57 |
| cation transmembrane transport | -0.57 |
| peptidoglycan biosynthetic process | -0.53 |
| detoxification of mercury ion | -0.52 |
| cation transport | -0.52 |
| protein peptidyl-prolyl isomerization | -0.51 |
| regulation of cell shape | -0.51 |
| biotin biosynthetic process | -0.50 |
| transcription-coupled nucleotide-excision repair, DNA damage recognition | -0.49 |
| DNA replication | -0.49 |
| cobalamin transport | -0.48 |
| RNA phosphodiester bond hydrolysis | -0.48 |
| rRNA methylation | -0.47 |
| tRNA 3'-terminal CCA addition | -0.47 |
| RNA repair | -0.47 |
| protein retention in ER lumen | -0.46 |
| manganese ion transmembrane transport | -0.46 |
| tetrahydrobiopterin biosynthetic process | -0.46 |
| nucleic acid phosphodiester bond hydrolysis | -0.45 |
| DNA strand renaturation | -0.45 |
| response to mercury ion | -0.45 |
| cobalamin biosynthetic process | -0.45 |
| lipopolysaccharide metabolic process | -0.44 |
| regulation of lipid biosynthetic process | -0.44 |
| cellular manganese ion homeostasis | -0.44 |
| phosphorus metabolic process | -0.43 |
| tRNA guanine ribose methylation | -0.43 |
| photosystem II stabilization | -0.43 |
| rhythmic process | -0.43 |
| response to UV | -0.42 |
| negative regulation of translation | -0.42 |
| DNA repair | -0.42 |
| signal transduction by protein phosphorylation | -0.42 |
| rRNA 2'-O-methylation | -0.42 |
| regulation of DNA repair | -0.41 |
| tRNA pseudouridine synthesis | -0.41 |
| photosynthesis | -0.41 |
| cellular protein modification process | -0.41 |
| maintenance of CRISPR repeat elements | -0.41 |
| RNA methylation | -0.41 |
| DNA metabolic process | -0.41 |
| replication fork processing | -0.41 |
| pyrimidine deoxyribonucleoside triphosphate catabolic process | -0.40 |
| dITP catabolic process | -0.40 |
| uracil transport | -0.40 |
| uracil transmembrane transport | -0.40 |
| DNA replication, synthesis of RNA primer | -0.40 |

Table S5. Gene Ontology (GO) biological categories that significantly correlated (Mantel test statistic, *p*-value < 0.05) to the total organic carbon distribution (TOC).

| **Biological functions** | **positive r** |
| --- | --- |
| purine-containing compound salvage | 0.62 |
| xanthine metabolic process | 0.62 |
| fumarate transport | 0.61 |
| succinate transmembrane transport | 0.61 |
| pentose metabolic process | 0.60 |
| NADP metabolic process | 0.56 |
| L-asparagine biosynthetic process | 0.54 |
| lipoprotein metabolic process | 0.53 |
| branched-chain amino acid catabolic process | 0.52 |
| 2,4-dichlorophenoxyacetic acid catabolic process | 0.51 |
| valine catabolic process | 0.51 |
| alpha-glucan catabolic process | 0.49 |
| cellular oligosaccharide catabolic process | 0.49 |
| alkaloid metabolic process | 0.48 |
| response to drug | 0.47 |
| tRNA 3'-trailer cleavage, endonucleolytic | 0.47 |
| xenobiotic catabolic process | 0.47 |
| peptide metabolic process | 0.47 |
| rhamnose transmembrane transport | 0.47 |
| ferulate metabolic process | 0.47 |
| cinnamic acid catabolic process | 0.47 |
| leucine catabolic process | 0.46 |
| regulation of viral transcription | 0.45 |
| actin filament bundle assembly | 0.45 |
| cholesterol biosynthetic process | 0.45 |
| histone modification | 0.44 |
| glycerophosphodiester transmembrane transport | 0.44 |
| amino-acid betaine catabolic process | 0.43 |
| organic acid transport | 0.43 |
| xenobiotic transport | 0.43 |
| sodium ion export across plasma membrane | 0.43 |
| potassium ion export across plasma membrane | 0.43 |
| aggregation involved in sorocarp development | 0.43 |
| autophagic cell death | 0.43 |
| D-xylose transmembrane transport | 0.43 |
| locomotion | 0.43 |
| hemolysis by symbiont of host erythrocytes | 0.43 |
| cellular monovalent inorganic cation homeostasis | 0.43 |
| response to osmotic stress | 0.43 |
| cell-substrate adhesion | 0.42 |
| peptidyl-lysine demalonylation | 0.42 |
| peptidyl-lysine desuccinylation | 0.42 |
| 4-hydroxyphenylacetate catabolic process | 0.42 |
| negative regulation of Wnt signaling pathway | 0.42 |
| glucose transmembrane transport | 0.42 |
| response to extracellular stimulus | 0.41 |
| gene expression | 0.41 |
| actin filament depolymerization | 0.41 |
| regulation of myosin II filament disassembly | 0.41 |
| mitotic cleavage furrow formation | 0.41 |
| intranuclear rod assembly | 0.41 |
| cellular response to acidic pH | 0.41 |
| cytokine-mediated signaling pathway | 0.41 |
| galactitol transport | 0.41 |
| aspartate biosynthetic process | 0.41 |
| antibiotic catabolic process | 0.40 |
| cellular amino acid catabolic process | 0.40 |
| tRNA 3'-trailer cleavage | 0.40 |
| **Biological functions** | **negative r** |
| cation transmembrane transport | -0.57 |
| tRNA seleno-modification | -0.55 |
| detoxification of mercury ion | -0.53 |
| pyrimidine deoxyribonucleoside triphosphate catabolic process | -0.53 |
| dITP catabolic process | -0.53 |
| D-ribose catabolic process | -0.50 |
| cation transport | -0.50 |
| response to mercury ion | -0.50 |
| mitochondrial ATP synthesis coupled proton transport | -0.50 |
| RNA phosphodiester bond hydrolysis | -0.49 |
| DNA replication | -0.49 |
| DNA replication, synthesis of RNA primer | -0.49 |
| regulation of cell shape | -0.48 |
| transcription-coupled nucleotide-excision repair, DNA damage recognition | -0.48 |
| DNA strand renaturation | -0.47 |
| tetrahydrobiopterin biosynthetic process | -0.47 |
| cobalamin biosynthetic process | -0.47 |
| tRNA 3'-terminal CCA addition | -0.46 |
| RNA repair | -0.46 |
| peptidoglycan biosynthetic process | -0.45 |
| cellular protein modification process | -0.45 |
| cobalamin transport | -0.45 |
| protein retention in ER lumen | -0.45 |
| tRNA pseudouridine synthesis | -0.45 |
| negative regulation of translation | -0.44 |
| response to UV | -0.44 |
| DNA metabolic process | -0.44 |
| anaphase-promoting complex-dependent catabolic process | -0.44 |
| protein polymerization | -0.44 |
| dUTP biosynthetic process | -0.43 |
| purine ribonucleotide biosynthetic process | -0.43 |
| photosynthesis | -0.43 |
| lipopolysaccharide metabolic process | -0.43 |
| regulation of lipid biosynthetic process | -0.43 |
| replication fork processing | -0.43 |
| ribosome biogenesis | -0.42 |
| plasmid maintenance | -0.42 |
| uracil transport | -0.42 |
| uracil transmembrane transport | -0.42 |
| spindle assembly | -0.41 |
| protein transport by the Tat complex | -0.41 |
| toxin biosynthetic process | -0.41 |
| rRNA methylation | -0.40 |
| folic acid-containing compound biosynthetic process | -0.40 |
| D-ribose metabolic process | -0.40 |
| photosystem II stabilization | -0.40 |
| RNA methylation | -0.40 |
| regulation of DNA repair | -0.40 |

Table S6. Gene Ontology (GO) biological categories that significantly correlated (Mantel test statistic, *p*-value < 0.05) to the total nitrogen distribution (TN).

| **Biological functions** | **positive r** |
| --- | --- |
| oxalate metabolic process | 0.64 |
| organic phosphonate catabolic process | 0.64 |
| XMP salvage | 0.62 |
| nitrogen compound metabolic process | 0.58 |
| oligosaccharide metabolic process | 0.57 |
| chromate transport | 0.57 |
| leucine biosynthetic process | 0.57 |
| histone modification | 0.56 |
| glucose transmembrane transport | 0.56 |
| carnitine biosynthetic process | 0.55 |
| protein ubiquitination | 0.54 |
| phytochelatin biosynthetic process | 0.53 |
| copper ion homeostasis | 0.51 |
| branched-chain amino acid metabolic process | 0.51 |
| nucleoside transmembrane transport | 0.51 |
| urea metabolic process | 0.50 |
| branched-chain amino acid catabolic process | 0.50 |
| D-gluconate metabolic process | 0.49 |
| poly-hydroxybutyrate biosynthetic process | 0.49 |
| lipoprotein metabolic process | 0.49 |
| purine-containing compound salvage | 0.49 |
| xanthine metabolic process | 0.49 |
| inositol biosynthetic process | 0.49 |
| organic phosphonate metabolic process | 0.48 |
| carbohydrate transport | 0.47 |
| arabinose metabolic process | 0.47 |
| leucyl-tRNA aminoacylation | 0.47 |
| cellular aromatic compound metabolic process | 0.47 |
| arginine biosynthetic process | 0.47 |
| 2-aminoethylphosphonate transport | 0.47 |
| aerobic respiration | 0.47 |
| transmembrane transport | 0.46 |
| D-xylose metabolic process | 0.46 |
| karyogamy involved in conjugation with cellular fusion | 0.46 |
| dicarboxylic acid transport | 0.45 |
| Actinobacterium-type cell wall biogenesis | 0.45 |
| CENP-A containing chromatin organization | 0.45 |
| Okazaki fragment processing involved in mitotic DNA replication | 0.45 |
| regulation of DNA double-strand break processing | 0.45 |
| regulation of histone H2B conserved C-terminal lysine ubiquitination | 0.45 |
| anaerobic ethylbenzene catabolic process | 0.45 |
| aromatic compound catabolic process | 0.45 |
| monosaccharide transmembrane transport | 0.45 |
| polyamine transport | 0.45 |
| protein-containing complex assembly | 0.44 |
| protein secretion by the type IV secretion system | 0.44 |
| positive regulation of GTPase activity | 0.44 |
| D-xylose transmembrane transport | 0.44 |
| actin filament organization | 0.44 |
| valine catabolic process | 0.44 |
| teichoic acid biosynthetic process | 0.44 |
| arginine biosynthetic process via ornithine | 0.44 |
| histone H3-K79 methylation | 0.44 |
| fungal-type cell wall beta-glucan biosynthetic process | 0.44 |
| ribosomal large subunit biogenesis | 0.44 |
| response to unfolded protein | 0.44 |
| S-adenosylmethionine biosynthetic process | 0.43 |
| intracellular signal transduction | 0.43 |
| regulation of circadian rhythm | 0.43 |
| urea catabolic process | 0.43 |
| posttranslational protein targeting to membrane, translocation | 0.43 |
| chromatin remodeling | 0.43 |
| xenobiotic transport | 0.43 |
| protein import into nucleus | 0.43 |
| glutamate biosynthetic process | 0.42 |
| cellular response to starvation | 0.42 |
| sodium-dependent phosphate transport | 0.42 |
| glycosaminoglycan metabolic process | 0.42 |
| gamma-aminobutyric acid transport | 0.42 |
| establishment or maintenance of cell polarity regulating cell shape | 0.42 |
| maturation of 5.8S rRNA from tricistronic rRNA transcript | 0.42 |
| lipid transport | 0.42 |
| glyoxylate catabolic process | 0.42 |
| microtubule-based movement | 0.42 |
| molybdate ion transport | 0.42 |
| enzyme active site formation | 0.42 |
| nucleoside catabolic process | 0.42 |
| cell cycle checkpoint | 0.42 |
| response to drug | 0.42 |
| ethanolamine catabolic process | 0.42 |
| mRNA splicing, via spliceosome | 0.41 |
| polyketide metabolic process | 0.41 |
| beta-lactam antibiotic catabolic process | 0.41 |
| gluconate transmembrane transport | 0.41 |
| cell adhesion | 0.41 |
| fucose transmembrane transport | 0.41 |
| glutamine biosynthetic process | 0.41 |
| formaldehyde catabolic process | 0.41 |
| regulation of transcription by RNA polymerase II | 0.41 |
| chromatin silencing at centromere | 0.41 |
| nitrogen compound transport | 0.41 |
| histone deacetylation | 0.41 |
| ubiquitin-dependent protein catabolic process | 0.40 |
| response to methotrexate | 0.40 |
| small GTPase mediated signal transduction | 0.40 |
| **Biological functions** | **negative r** |
| semaphorin-plexin signaling pathway | -0.55 |
| protein adenylylation | -0.48 |
| DNA double-strand break processing | -0.48 |
| intra-S DNA damage checkpoint | -0.48 |
| N-acylethanolamine metabolic process | -0.45 |
| reductive pentose-phosphate cycle | -0.45 |
| U2-type prespliceosome assembly | -0.44 |
| maintenance of rDNA | -0.44 |
| resolution of mitotic recombination intermediates | -0.44 |
| regulation of mitotic recombination involved in replication fork processing | -0.44 |
| enterobacterial common antigen biosynthetic process | -0.44 |
| photorespiration | -0.43 |
| arginine catabolic process to glutamate | -0.43 |
| carbon fixation | -0.42 |
| carboxylic acid transmembrane transport | -0.42 |
| negative regulation of TOR signaling | -0.41 |
| cellular response to amino acid starvation | -0.41 |
| arginine catabolic process to succinate | -0.40 |
| rRNA (guanine-N7)-methylation | -0.40 |
